# Quantitative understanding of PDF fits and their uncertainties

**DOI:** 10.1140/epjc/s10052-026-16008-0

**Published:** 2026-06-28

**Authors:** Amedeo Chiefa, Luigi Del Debbio, Richard Kenway

**Affiliations:** The Higgs Centre for Theoretical Physics, School of Physics and Astronomy, The University of Edinburgh, Peter Guthrie Tait Road, Edinburgh, EH9 3FD UK

## Abstract

Parton distribution functions (PDFs) play a central role in describing experimental data at colliders and provide insight into the structure of nucleons. As the LHC enters an era of high-precision measurements, a robust PDF determination with a reliable uncertainty quantification has become mandatory in order to match the experimental precision. The NNPDF collaboration has pioneered the use of machine learning (ML) techniques for PDF determinations, using neural networks (NNs) to parametrise the unknown PDFs in a flexible and unbiased way. The NNs are then trained on experimental data by means of stochastic gradient descent algorithms. The statistical robustness of the results is validated by extensive closure tests using synthetic data. In this work, we develop a theoretical framework based on the neural tangent kernel (NTK) to analyse the training dynamics of neural networks. This approach allows us to derive, under precise assumptions, an analytical description of the neural network evolution during training, enabling a quantitative understanding of the training process. Having an analytical handle on the training dynamics allows us to clarify the role of the NN architecture and the impact of the experimental data in a transparent way. Similarly, we are able to describe the evolution of the covariance of the NN output during training, providing a quantitative description of how uncertainties are propagated from the data to the fitted function. Interestingly, the methodology developed in this work can be used to understand the minimization of a loss function for any kind of parametrization, thereby providing a unified framework to compare different PDF determinations, like, e.g., fits based on a particular functional form. While our results are *not* a substitute for PDF fitting, they do provide a powerful diagnostic tool to assess the robustness of current fitting methodologies. Beyond its relevance for particle physics phenomenology, our analysis of PDF determinations provides a testbed to apply theoretical ideas about the learning process developed in the ML community. As seen in applications from other domains, we find that our results deviate from the simple picture of the *lazy training* regime discussed in the ML literature.

## Introduction

Parton distribution functions (PDFs) are a central ingredient in describing experimental data at hadron colliders and in gaining insights into the internal structure of the proton. The high-precision era of particle physics that we are now witnessing calls for equally precise theoretical predictions. Since PDFs are a key ingredient in these predictions, the need for robust PDF determinations with reliable uncertainty quantification has become increasingly important for both Standard Model measurements and searches for new physics.

PDFs are typically extracted from global analyses of experimental and lattice data. Their determination is a classic example of an *inverse problem*, as it involves inferring a continuous function from a finite set of data points. This process is inherently ill-defined, and the limited amount of experimental information prevents us from obtaining a unique solution to the problem. The solution will inevitably depend on the assumptions made and on the prior knowledge introduced to regularise the problem, either explicitly stated or implicitly embedded in the fitting framework.

The complex nature of inverse problems has prompted the development of sophisticated statistical methods and tools to tackle them. In general, PDF determinations can be broadly classified into two main categories, depending on whether a specific functional form is assumed for the PDFs or whether a non-parametric approach is adopted. Although the former approach has been widely used in the literature, non-parametric approaches based on Bayesian inference have been successfully applied to the problem of PDF determination, albeit in a limited scenario [1–3]. Bayesian-based approaches are promising tools that ensure a rigorous framework where prior information and assumptions are spelled out explicitly. Yet, a global PDF determination based on these methods has not yet been attempted, and the impact of the prior needs to be carefully studied in these frameworks.

On the other hand, state-of-the-art PDF determinations rely on parametric approaches, where a specific functional form is assumed for the PDFs at a given initial scale $Q_0$. These functions are typically chosen to be flexible enough to capture the main features of the PDFs, while their internal parameters are optimised to reproduce the experimental data. Several groups [4–7] have set the standard for PDF determinations through continuous refinement of their global fits as new data and theoretical advances become available, with an increasing emphasis on uncertainty quantification. Although these determinations have been shown to perform well on a wide range of new experimental data [8], the different methodological frameworks adopted by the various groups lead to PDF sets whose differences are yet to be fully understood [9, 10]. These differences become significantly visible when considering parameter determinations that are particularly sensitive to the choice of the PDF set, both on the central values and, most importantly, on the associated uncertainties (see Refs. [11–14] for some recent examples).

In this work, we build upon the work of Refs. [1, 2], which aims at providing a sound statistical framework for PDF determination, with all underlying assumptions clearly stated. We focus on the NNPDF methodology [4], which pioneered the use of ML tools in the context of PDF determinations and has been validated through extensive studies over the years [1, 15, 16]. It combines a Monte Carlo sampling of the experimental data and a feed-forward neural network parametrization of the PDFs. We adopt a simplified framework to analyse the training process, aiming at providing a quantitative description of its key aspects, and making transparent the assumptions that are often implicitly embedded in the fitting procedure.

We demonstrate that the training dynamics of a neural network can be fully reformulated in functional space, leading to an interpretable description of the learning process. We show that the training dynamics is dictated by the Neural Tangent Kernel (NTK) [17], which encodes and factorises the dependence on the architecture and the parameters of the neural network. Similar approaches leveraging NTK properties have been explored in other contexts [18]. In fact, the spectral properties of the NTK provide a powerful lens through which we can understand the learning process: only the directions that are orthogonal to the kernel of the NTK are actually learned in the training process. At initialisation, the NTK is characterised by a wide spectrum of eigenvalues, with only a few large eigenvalues being significantly different from zero. Even though the NNs span a very broad functional space, the training explores a much smaller subspace. During the training process, the hierarchy in the NTK spectrum is preserved, but eigenvalues that were initially subleading, or zero, grow in magnitude. Since the only directions that contribute to the learning process are those associated to the non-zero eigenvalues, with the actual value of the eigenvalue setting the learning speed along the corresponding eigenvector direction, the growth of some eigenvalues indicates that new features in the functional space emerge during training and that the network thus becomes capable of representing more complex functions. The space of functions explored in the training process is therefore dynamically determined during the training itself, exploiting the flexibility of the parametrization in order to explore multiple functional forms and select the preferred one based on inference from data rather than a priori decisions.

Another key result of this work is that, after an initial transient phase where the NTK evolves significantly, the training process enters a second regime where the NTK becomes approximately constant. This regime is often referred to as *lazy training* in the machine learning literature [17], and it has important implications for the training dynamics. In this regime, we show that the training process can be described analytically, allowing us to obtain a single and clean closed-form expression for the output of the network at any training time *t*. The main result of this analysis is summarised in Eq. (60) which we report here,

$$ f_{t} = U(t) f_{0} + V(t) Y\, , $$

where $f_{t}$ is the network output at training time *t*, $f_{0}$ is the initial output at $t=0$, *Y* are the training data, and *U*(*t*) and *V*(*t*) are time-dependent matrices that depend on the NTK and are computed explicitly in Sect. 4. It is interesting to remark that this expression decomposes into two contributions: one that depends on the initial condition and another that depends on the data, thus making explicit the role of prior information and of experimental measurements in shaping the final result. This analytical expression also allows us to compute the evolution of the covariance of the network output during training, providing a quantitative description of how uncertainties are propagated from the data to the fitted function. Although applicable only when the NTK reaches stability, this analytical description is a powerful tool to bridge the gap between the parametric regression approach adopted in NNPDF and other methods for solving inverse problems that are receiving growing attention in the community.

Being derived in a simplified setting – considering a single PDF flavor combination with DIS data and vanilla gradient descent optimization – we present this study as an exploration of foundational aspects, primarily focussed on the theoretical issues; further investigations are in progress in order to extend these ideas to the full complexity of modern global PDF fits. We particularly emphasise that the present analysis is not limited to neural networks, but can be extended to any functional parametrization that undergoes a gradient-based training process. It will be interesting to explore the properties of the NTK together with its spectral structure in more realistic PDF fits, translating the differences between various fitting methodologies in terms of the NTK. We leave these studies to future work.

The remainder of this paper is organized as follows. In Sect. 2 the inverse problem of PDF determination is briefly reviewed in the simplified case of theoretical predictions that depend linearly on the PDFs. We then review some fundamental statistical aspects of the Neural Networks at initialisation, which will be relevant in the rest of the paper. The training dynamics is then discussed in Sect. 3, where the learning process of the neural network is reformulated in functional space by means of the NTK. The implications of the *lazy training* regime are used in Sect. 4 to derive an analytical description of the training process. Finally, we present our conclusions and outlook in Sect. 5.

## Neural networks and PDFs

In the following, we prepare the ground for the study of the training dynamics of neural networks used in the NNPDF framework. We start by briefly presenting the inverse problem of PDF determination using data depending linearly on the PDFs, setting the notation and introducing the statistical vocabulary used in the rest of this study. We then discuss some statistical aspects of the neural networks at initialisation, which will help us understand the implications in the training process. These properties, derived in the large-width limit [17, 19], are analysed for the specific architecture used in the NNPDF methodology. An exhaustive and detailed review of wide-network properties is beyond the scope of this work, and the reader is encouraged to refer to Ref. [20] for a comprehensive review.

### The 1-dimensional regression problem of PDFs

The extraction of PDFs from experimental data is a classic example of an inverse problem, namely the reconstruction of a function *f*(*x*) from a finite set of data points $Y_I$, where the index $I=1, \ldots , N_{\textrm{dat}}$.^1^ In particular, for this study, we will focus on DIS data, which depend linearly on the function *f*(*x*). The theoretical prediction for the data point $Y_I$ is given by

1 $$\begin{aligned} T_I[f] = \sum _{i=1}^{N_{f}} \int dx\, C_{Ii}(x) f_{i}(x)\,, \end{aligned}$$

where $C_{Ii}(x)$ is a coefficient function, known to some given order in perturbation theory, $i = 1, \ldots , N_{f}$, labels the parton flavor, and $f_i(x)$ is the PDF (or set of PDFs) that we want to determine.

Attempting to determine a function *f* in an infinite dimensional space of solutions using a finite set of data is inherently ill-posed. The solution inevitably depends on assumptions and prior knowledge – conscious or not – introduced to regularise the problem. Different methodologies, based either on non-parametric methods or parametric regression, have been proposed to address these challenges, yielding increasingly precise PDFs. Yet, despite the longstanding effort to provide robust uncertainty quantification and establish the relationships between different methodologies and their solutions, some discrepancies remain unresolved, see, e.g., [10]. Understanding such differences between the various approaches is thus crucial for precision physics.

Following the ideas highlighted in Refs. [1, 2], the solution of the inverse problem is conveniently phrased in a Bayesian framework. The functions $f_i$ are promoted to stochastic processes; for any grid of points $x_{\alpha }$, $\alpha =1, \ldots , N_{\textrm{grid}}$, the vector $f_{i\alpha }=f_{i}(x_{\alpha })$ is a vector of $N_{f}\times N_{\textrm{grid}}$ stochastic variables, for which we introduce a *prior* distribution *p*(*f*).^2^ In this perspective, any fitting procedure is interpreted as a recipe that yields the *posterior* distribution $\tilde{p}(f) = p(f | Y)$. In this study, following the NNPDF methodology, probability distributions are represented by ensembles of i.i.d. neural network replicas. So, for instance, the prior distribution *p*(*f*) is described by an ensemble

2 $$\begin{aligned} \left\{ f^{(k)} \in \mathbb {R}^{N_{f}\times N_{\textrm{grid}}}; k=1, \ldots , N_\textrm{rep}\right\} \,, \end{aligned}$$

drawn from the distribution *p*, so that

3 $$\begin{aligned} \mathbb {E}_{p}[O(f)] = \frac{1}{N_\textrm{rep}} \sum _{k=1}^{N_\textrm{rep}} O(f^{(k)})\,, \end{aligned}$$

for any observable *O* that is built from the PDFs.

The prior distribution *p*(*f*) is defined by initializing a set of neural networks (NNs) replicas using a Glorot normal initializer [21]. The result of this initialisation is discussed below in Sect. 2.2.

In order to account for the experimental uncertainties and propagate them to the fitted PDFs, the NNPDF collaboration uses Monte Carlo replicas [22]. For each replica, labelled by the index *k*, a new set of data $Y^{(k)}$ is generated from an $N_{\textrm{dat}}$ dimensional Gaussian distribution centred at the experimental central value *Y*, with the covariance given by the experimental covariance matrix $C_Y$,

4 $$\begin{aligned} Y^{(k)} \sim \mathcal {N}\left( Y, C_Y\right) \,. \end{aligned}$$

Each replica $f^{(k)}$ is trained on its corresponding data set $Y^{(k)}$. We denote the replicas at training time *t* as $f^{(k)}_{t} \in \mathbb {R}^{N_{f}\times N_{\textrm{grid}}}$. Stopping the training at time *T*, the posterior probability distribution is represented by the set of *trained* replicas $\left\{ f^{(k)}_{T}\in \mathbb {R}^{N_{f}\times N_{\textrm{grid}}}; k=1, \ldots , N_\textrm{rep}\right\}$, so that averages over the posterior distribution are computed as

5 $$\begin{aligned} \mathbb {E}_{\tilde{p}}[O(f)] = \frac{1}{N_\textrm{rep}} \sum _{k=1}^{N_\textrm{rep}} O\left( f^{(k)}_{T}\right) \,. \end{aligned}$$

All knowledge about the solution of the inverse problem, *f*, is encoded in the posterior $\tilde{p}$ and is expressed as expectation values of observables *O* using Eq. (5). Let us stress once again that the expectation values with respect to the prior and posterior distributions are both obtained by taking averages over replicas. The expectation value with respect to the prior is the average over replicas at initialization. The expectation value with respect to the posterior is the average over the replicas at training time *T*.

Training may yield different posteriors depending on the initial network configuration. To understand this dependence, we pause to examine the statistical properties of network ensembles at initialization. This analysis provides a quantitative insight into how prior knowledge embedded in the initialization interacts with, and evolves throughout, the training process, as we show in Sect. 4.

### Neural networks at initialisation

When initializing a neural network, the weights and biases – which we denote collectively as the *parameters* of the network – are drawn from some probability distribution. In the NNPDF formalism, the set of network parameters at initialisation for each replica is an instance of i.i.d. stochastic variables. More importantly, the probability distribution of the network parameters induces a probability distribution for the output of the neural networks at initialisation. It is well known that the probability distribution of these outputs becomes approximately Gaussian when the size of the hidden layers is increased  [20]. We call this limit the *large-network* limit.

As detailed in Ref. [4], the NNs used for the NNPDF fit have a 2-25-20-8 architecture, a $\tanh$ activation function, and are initialized using a Glorot normal distribution [21]. The preactivation function of a neuron is denoted as $\phi ^{(\ell )}_{i,\alpha } = \phi ^{(\ell )}_i(x_\alpha )$, where $\ell = 0, \ldots , L$, denotes the layer of the neuron, and, for each $\ell$, $i=1, \ldots , n_{\ell }$ identifies the neuron within the layer.^3^ Furthermore, $x_{\alpha }$ is the input to the NN, i.e., a point in the interval [0, 1]. A grid of $N_{\textrm{grid}}=50$ points in *x* is used to compute observables in the NNPDF formalism and in this work we focus on the values of *f* at those values of $x_\alpha$, where the index $\alpha = 1, \ldots , N_{\textrm{grid}}$ labels the points on the grid. For completeness, we list the values of $x_\alpha$ in Table 1.

**Table 1:** Values of $x_\alpha$ used in the NNPDF grids for the computation of observables. The points are equally spaced on a logarithmic scale for $\alpha = 1, \ldots , 27$, and linearly spaced for $\alpha > 27$

$\alpha$	$x_\alpha$	$\alpha$	$x_\alpha$	$\alpha$	$x_\alpha$	$\alpha$	$x_\alpha$	$\alpha$	$x_\alpha$
1	$2.00 \times 10^{-7}$	11	$1.29 \times 10^{-5}$	21	$8.31 \times 10^{-4}$	31	0.0434	41	0.422
2	$3.03 \times 10^{-7}$	12	$1.96 \times 10^{-5}$	22	$1.26 \times 10^{-3}$	32	0.0605	42	0.480
3	$4.60 \times 10^{-7}$	13	$2.97 \times 10^{-5}$	23	$1.90 \times 10^{-3}$	33	0.0823	43	0.540
4	$6.98 \times 10^{-7}$	14	$4.51 \times 10^{-5}$	24	$2.87 \times 10^{-3}$	34	0.109	44	0.601
5	$1.06 \times 10^{-6}$	15	$6.84 \times 10^{-5}$	25	$4.33 \times 10^{-3}$	35	0.141	45	0.665
6	$1.61 \times 10^{-6}$	16	$1.04 \times 10^{-4}$	26	$6.50 \times 10^{-3}$	36	0.178	46	0.730
7	$2.44 \times 10^{-6}$	17	$1.57 \times 10^{-4}$	27	$9.70 \times 10^{-3}$	37	0.220	47	0.796
8	$3.70 \times 10^{-6}$	18	$2.39 \times 10^{-4}$	28	0.0144	38	0.265	48	0.863
9	$5.61 \times 10^{-6}$	19	$3.62 \times 10^{-4}$	29	0.0211	39	0.314	49	0.931
10	$8.52 \times 10^{-6}$	20	$5.49 \times 10^{-4}$	30	0.0305	40	0.367	50	1.00

The output of the neuron is identified by the pair $(\ell ,i)$ is $\rho ^{(\ell )}_{i\alpha } = \tanh \left( \phi ^{(\ell )}_{i\alpha }\right)$. The parameters of the NN are the weights $w^{(\ell )}_{ij}$ and the biases $b^{(\ell )}_i$, which are collectively denoted as $\theta _\mu$, where $\mu = 1, \ldots , P$, and the total number of parameters is

6 $$\begin{aligned} P = \sum _{\ell =1}^{L} \left( n_{\ell } n_{\ell -1} + n_\ell \right) \,. \end{aligned}$$

The preactivation function in layer $(\ell +1)$ is a weighted average of the outputs of the neurons on the previous layer, namely

7 $$\begin{aligned} \phi ^{(\ell +1)}_{i\alpha } = \sum _{j=1}^{n_\ell } w^{(\ell +1)}_{ij} \rho ^{(\ell )}_{j\alpha } + b^{(\ell +1)}_{i}\, . \end{aligned}$$

The PDFs in the so-called evolution basis are parametrized by the preactivation functions of the output layer *L*, $x_\alpha f_i(x_\alpha )= \phi ^{(L)}_{i,\alpha }$, where the neuron index on the last layer, $i=1, \ldots , 8$, labels the flavors.^4^ The input layer is identified by $\ell =0$ and the activation function for that specific layer is the identity, so that

8 $$\begin{aligned} \rho ^{(0)}_{i,\alpha } = \phi ^{(0)}_{i,\alpha } = x_{i,\alpha } = {\left\{ \begin{array}{ll} x_\alpha \,, \quad & \text {for}\ i=1\,;\\ \log \left( x_\alpha \right) \,, \quad & \text {for}\ i=2\,. \end{array}\right. } \end{aligned}$$

In the following we refer to the preactivation functions as *fields*. In Eq. (8), each input *x* is augmented with its logarithm, so that the vector $(x, \log x)$ is fed into the first hidden layer. This transformation, used in the NNPDF fits and referred to as “scaled inputs”, gives the network direct access to both linear and logarithmic scales of the input. Although the *x*-values are already sampled on a mixed logarithmic/linear grid (see Table 1), with a logarithmic spacing in the small-*x* region and a linear spacing in the large-*x* region, feeding $\log x$ explicitly allows the network to resolve PDF features across the full *x*-range more efficiently.

**Fig. 1:**
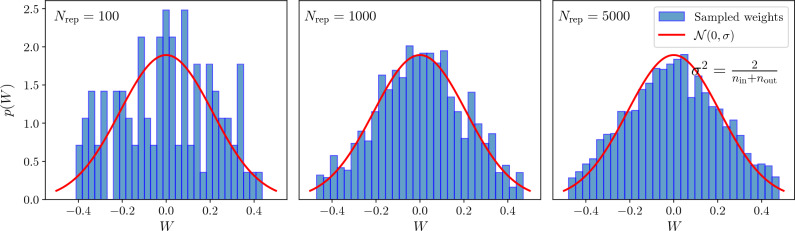
Sampled distribution of a selected weight as a function of the number of replicas. The red line represents the underlying Gaussian distribution from which the weights are drawn. As the number of replicas is increased the distribution of the weight converges to the expected Gaussian

The Glorot normal initialiser draws each weight and bias of the NN from independent Gaussian distributions, denoted $p_w$ and $p_b$ respectively, centred at zero and with variances rescaled by the number of nodes in adjacent layers,

9 $$\begin{aligned} \frac{C^{(\ell )}_{w}}{n_{\ell -1} + n_{\ell }}\,, \quad \frac{C^{(\ell )}_{b}}{n_{\ell -1} + n_{\ell }}\,. \end{aligned}$$

Following the NNPDF prescription, we have $C^{(\ell )}_{w}=C^{(\ell )}_{b}=1$. Figure 1 shows the binned distribution of one of the weights in the network as a function of the number of replicas. Together with the histogram, the underlying Gaussian, as dictated by the Glorot normal initialisation, is also shown. The figure illustrates how the distribution of the weights converges to the expected Gaussian as the number of replicas increases.

The probability distribution of the NN parameters induces a probability distribution for the preactivations; the probability distribution of the fields in layer $\ell$, for given values of the field in the layer $\ell -1$ is

10 $$\begin{aligned} p\left( \phi ^{(\ell )} | \phi ^{(\ell -1)}\right)&= \int \mathcal {D}w\, p_w(w)\, \mathcal {D}b\, p_b(b)\, \prod _{i,\alpha } \delta \nonumber \\&\quad \left( \phi ^{(\ell )}_{i\alpha } - \sum _{j} w^{(\ell )}_{ij} \rho \left( \phi ^{(\ell -1)}_{j\alpha }\right) - b^{(\ell )}_i \right) \, . \end{aligned}$$

For clarity of writing, we will omit the condition in the probability distribution, and write simply $p(\phi ^{(\ell )})$. Note that, here and in what follows, $p(\phi ^{(\ell )})$ denotes the joint probability for all the $n_{\ell }\times N_{\textrm{grid}}$ components of $\phi ^{(\ell )}$,

11 $$\begin{aligned} p\left( \phi ^{(\ell )}\right) = p\left( \phi ^{(\ell )}_{1,\alpha _1}, \phi ^{(\ell )}_{2,\alpha _1}, \ldots , \phi ^{(\ell )}_{n_\ell ,\alpha _1}, \phi ^{(\ell )}_{1,\alpha _2}, \ldots , \phi ^{(\ell )}_{n_\ell ,\alpha _2}, \ldots , \phi ^{(\ell )}_{n_\ell ,N_{\textrm{grid}}}\right) \, . \end{aligned}$$

This duality between parameter-space and function-space provides a powerful framework to study the behaviour of an ensemble of NNs, and in particular the symmetry properties of the distribution $p(\phi ^{(\ell )})$ (see, e.g., Ref. [23]). Working in parameter space, i.e., computing the expectation values of correlators of fields as integrals over the NN parameters, one can readily show that

12 $$\begin{aligned} \mathbb {E}\left[ R_{i_1j_1} \phi ^{(\ell )}_{j_1 \alpha _1} \ldots R_{i_nj_n} \phi ^{(\ell )}_{j_n \alpha _n} \right] = \mathbb {E}\left[ \phi ^{(\ell )}_{i_1 \alpha _1} \ldots \phi ^{(\ell )}_{i_n \alpha _n} \right] \, , \end{aligned}$$

where *R* is an orthogonal matrix in $\text {SO}(n_{\ell })$. Eq.(12) implies that the probability distribution in Eq. (10) is also invariant under rotations, and therefore it can only be a function of $\text {SO}(n_{\ell })$ invariants. Therefore

13 $$\begin{aligned} p\left( \phi ^{(\ell )}\right) = \frac{1}{Z^{(\ell )}} \exp \left( -S\left[ \phi ^{(\ell )}_{\alpha _1} \cdot \phi ^{(\ell )}_{\alpha _2}\right] \right) \, , \end{aligned}$$

where

14 $$\begin{aligned} \phi ^{(\ell )}_{\alpha _1} \cdot \phi ^{(\ell )}_{\alpha _2} = \sum _{i=1}^{n_\ell } \phi ^{(\ell )}_{i \alpha _1} \phi ^{(\ell )}_{i \alpha _2}\, . \end{aligned}$$

The action can be expanded in powers of the invariant bilinear,

15 $$\begin{aligned} S\left[ \phi ^{(\ell )}_{\alpha _1} \cdot \phi ^{(\ell )}_{\alpha _2}\right]&= \frac{1}{2} \gamma ^{(\ell )}_{\alpha _1\alpha _2} \phi ^{(\ell )}_{\alpha _1} \cdot \phi ^{(\ell )}_{\alpha _2} + \frac{1}{8 n_{\ell -1}} \gamma ^{(\ell )}_{\alpha _1\alpha _2,\alpha _3\alpha _4} \phi ^{(\ell )}_{\alpha _1}\nonumber \\&\quad \cdot \phi ^{(\ell )}_{\alpha _2} \, \phi ^{(\ell )}_{\alpha _3} \cdot \phi ^{(\ell )}_{\alpha _4} + O(1/n_{\ell -1}^2)\, , \end{aligned}$$

so that the probability distribution is fully determined by the couplings $\gamma ^{(\ell )}$.^5^ In Eq. (15), we have factored out inverse powers of $n_{\ell -1}$ for each coupling. With this convention, and with the scaling of the parameters variances in Eq. (9), the couplings in the action are all *O*(1) in the limit where $n_\ell \rightarrow \infty$. As a consequence, the probability distribution at initialisation is a multidimensional Gaussian at leading order – i.e., $\mathcal {O}(1)$ – in $1/n_\ell$, with quartic corrections that are $O(1/n_\ell )$, while higher powers of the invariant bilinear are suppressed by higher powers of the width of the layer. This power counting defines an effective field theory, where deviations from Gaussianity can be computed in perturbation theory to any given order in $1/n_\ell$, see, e.g.  Refs. [20, 24] for a detailed presentation of these ideas. While the actual calculations become rapidly cumbersome, the conceptual framework is straightforward.

At leading order, the second and fourth cumulant are respectively

16 $$\begin{aligned}&\langle \phi ^{(\ell )}_{i_1,\alpha _1} \phi ^{(\ell )}_{i_2,\alpha _2}\rangle = \delta _{i_1 i_2} K^{(\ell )}_{\alpha _1\alpha _2} + O(1/n_{\ell -1})\, , \end{aligned}$$

17 $$\begin{aligned}&\langle \phi ^{(\ell )}_{i_1,\alpha _1} \phi ^{(\ell )}_{i_2,\alpha _2} \phi ^{(\ell )}_{i_3,\alpha _3} \phi ^{(\ell )}_{i_4,\alpha _4}\rangle _c = O(1/n_{\ell -1})\, , \end{aligned}$$

where^6^

18 $$\begin{aligned} K^{(\ell )}_{\alpha _1\alpha _2} = \left( \gamma ^{(\ell )}\right) ^{-1}_{\alpha _1\alpha _2}\,. \end{aligned}$$

The “evolution” of the couplings as we go deep in the NN, i.e., the dependence of the couplings on $\ell$, is governed by Renormalization Group (RG) equations, which preserve the power counting in powers of $1/n_{\ell }$. At leading order,

19 $$\begin{aligned} K^{(\ell +1)}_{\alpha _1\alpha _2}&= \left. C_b^{(\ell +1)} + C_w^{(\ell +1)} \frac{n_\ell }{n_\ell +n_{\ell +1}}\frac{1}{n_\ell } \langle \vec {\rho }^{\,(\ell )}_{\alpha _1} \cdot \vec {\rho }^{\,(\ell )}_{\alpha _2} \rangle \right| _{O(1)} \end{aligned}$$

20 $$\begin{aligned}&= C_b^{(\ell +1)} + C_w^{(\ell +1)} \frac{n_\ell }{n_\ell +n_{\ell +1}}\frac{1}{n_\ell } \langle \vec {\rho }^{\,(\ell )}_{\alpha _1} \cdot \vec {\rho }^{\,(\ell )}_{\alpha _2} \rangle _{K^{(\ell )}}\, , \end{aligned}$$

where

$$\begin{aligned} \frac{1}{n_\ell } \langle \vec {\rho }^{\,(\ell )}_{\alpha _1} \cdot \vec {\rho }^{\,(\ell )}_{\alpha _2} \rangle _{K^{(\ell )}} = \int \mathcal {D}\phi \, \frac{e^{-\frac{1}{2} \left( K^{(\ell )}\right) ^{-1}_{\beta _1\beta _2} \phi _{\beta _1} \phi _{\beta _2}}}{\left| 2\pi K^{(\ell )}\right| ^{1/2}}\, \rho (\phi _{\alpha _1}) \rho (\phi _{\alpha _2})\, , \end{aligned}$$

and

21 $$\begin{aligned} \mathcal {D}\phi = \prod _{\alpha =1}^{N_{\textrm{grid}}} d\phi _\alpha \, . \end{aligned}$$

**Fig. 2:**
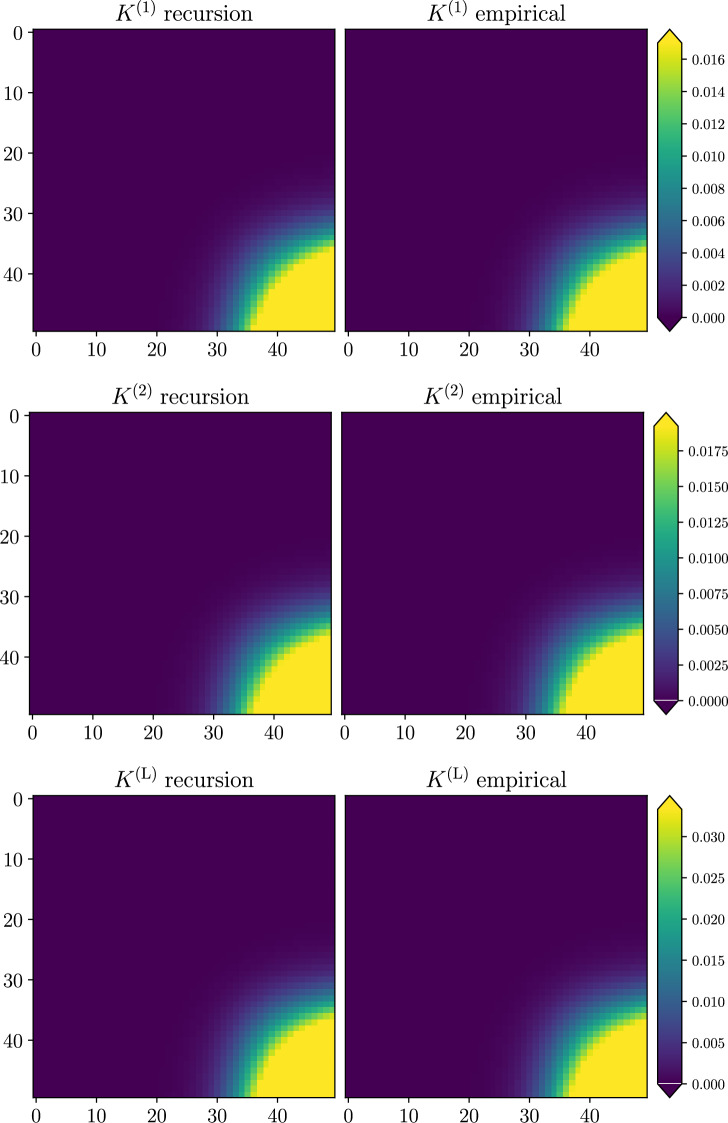
The empirical (left) and analytical (right) covariance matrices of the first, second and output layers of the NNPDF architecture (top to bottom). The covariance in the left panel is computed “bootstrapping” over an ensemble of replicas, initialised using the Glorot normal distribution. The covariance in the right panel is obtained by solving Eq. (20) numerically. In order to reduce the bootstrap errors in the empirical covariance, an ensemble of 1000 replicas has been used for this figure

**Fig. 3:**
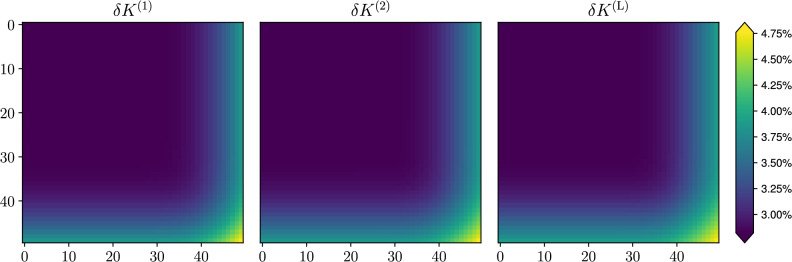
Relative difference between the empirical kernel, computed from an ensemble of networks at initialisation, and the recursive kernel obtained by iterating Eq. (20) for the three layers of the NNPDF architecture. An ensemble of 1000 replicas has been used to reduce the bootstrap errors in the empirical covariance

Note that the integration variables in Eq. (21) do not have a neuron index and the integrals are $N_{\textrm{grid}}$ dimensional integrals. Eq. (20) is iterated for the NNPDF architecture, yielding $K^{(\ell )}$ for arbitrary $\ell$, i.e., the covariance at initialisation for various depths. These are compared with the empirical covariance computed from an ensemble replicas in Fig. 2 for the first two hidden layers and the output layer. Furthermore, the relative difference between the empirical covariance and the theoretical prediction is shown in Fig. 3. In order to reduce the bootstrap errors in the empirical covariance, an ensemble with $N_\textrm{rep}=1000$ has been used for these figures. The agreement between the theoretical prediction and the empirical computation is excellent, confirming the validity of the large-network expansion even for networks of moderate size, as those used in the NNPDF fits.

**Fig. 4:**
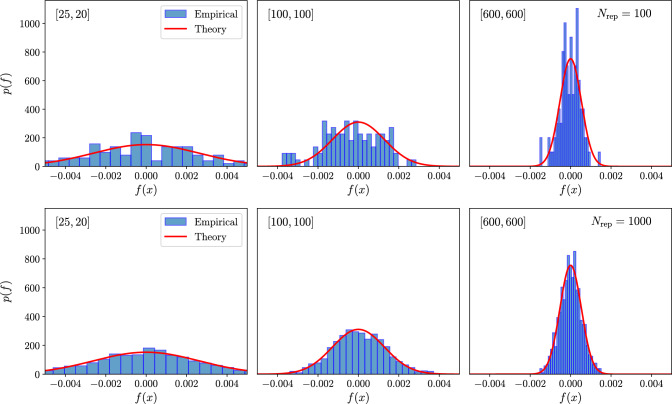
Sampled distribution of the output $xT_3$ at $x=0.0065$ for two different ensemble sizes, $N_\textrm{rep}=100$ (top) and $N_\textrm{rep}=1000$ (bottom). Each column shows the distribution for a different network architecture, the latter displayed in the top left corner of each panel. The red line represents the predicted Gaussian distribution as dictated by the kernel recursion formula in Eq. (20)

**Fig. 5:**
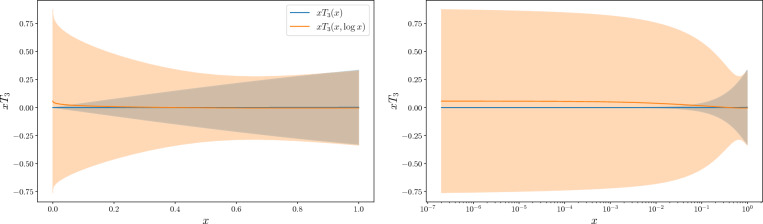
The output of the ensemble of neural networks at initialisation using the NNPDF architecture in linear (left) and logarithm (right) scale. We compare the case of linear input *f*(*x*) (blue) and the case of scaled input $f(x, \log x)$ (orange). The solid lines represent the mean value computed over an ensemble of 100 replicas, while the shaded bands represent the one-sigma uncertainty computed as the variance over the same ensemble. In the figure, we show $xT_3$ as used in the following sections

As a consequence of the symmetry of the probability distribution, the mean value of the fields at initialisation needs to vanish, while their variance at each point $x_\alpha$ is given by the diagonal matrix elements of $K^{(\ell )}$. In Fig. 4, the expected distribution is compared against the empirical distribution of output fields for a selected value of *x*, using two ensembles of replicas with $N_\textrm{rep}=100$ and $N_\textrm{rep}=1000$, respectively. Inspecting the figures, we conclude that the recursion formula, Eq. (20), accurately reproduces the output distribution of the NNPDF networks at initialisation, provided that a sufficiently large ensemble of replicas is used to sample the distribution. Finally, Fig. 5 shows the mean and variance of the output at initialisation across all values of *x* for an ensemble of $N_\textrm{rep}=100$ neural networks generated using the NNPDF architecture. We compare two cases: linear input *f*(*x*) and scaled input $f(x, \log x)$ as defined in Eq. (8). The central value is computed according to Eq. (3),

22 $$\begin{aligned} \bar{f}_{i\alpha } = \bar{f}_{i}(x_\alpha ) = \frac{1}{N_\textrm{rep}} \sum _{k=1}^{N_\textrm{rep}} f^{(k)}_i(x_\alpha )\, , \end{aligned}$$

and the variance $\sigma ^2_{i\alpha }$ is computed using the same formula with

23 $$\begin{aligned} O(f) = \frac{N_\textrm{rep}}{N_\textrm{rep}-1} \left( f_i(x_\alpha ) - \bar{f}_{i}(x_\alpha )\right) ^2\, . \end{aligned}$$

As is clear from the figure, the choice of input scaling has a significant impact of the prior uncertainty, especially in the small-*x* region. In the following, we neglect this effect and focus on the case of linear input *f*(*x*).

## Training dynamics and the neural tangent kernel

Having defined the physics goals and summarised some properties of the neural network at initialisation, we now turn to the optimisation process. In the context of machine learning, specifically when dealing with neural networks, optimisation is an iterative algorithm that updates the parameters of the network in order to minimise a figure of merit defined appropriately. Due to the large number of parameters that characterise a neural network, and the complex functional form induced by the recursive definition of the network, the figure of merit (also known as *error function*, *loss function*, or simply *loss*) is a non-convex high-dimensional function of the parameters, leading to numerical challenges in the minimisation task. In addition, in order to avoid *over-* and *under-learning*, training algorithms are complemented by a so-called *stopping criterion*, which specifies the optimal condition to end the training process.

In practice, minimization is performed using gradient methods where the direction towards the minimum is defined by the gradient of the loss function. These methods are usually improved by including, for instance, stochasticity and information on previous iterations [25–27]. A detailed overview of these extended gradient methods is beyond the scope of this work. In the context of PDF determinations, the NNPDF collaboration makes intensive use of these tools and the reader is encouraged to refer to Ref. [4] for an extensive discussion.

Our main aim in this paper is understanding the dynamics driving the training process. Indeed, while these algorithms have achieved remarkable empirical success, a theoretical understanding of the optimization process remains elusive. Therefore, we work with the simplest gradient method, i.e., gradient descent (GD). Furthermore, we consider a reduced dataset for which predictions can be computed using one flavor combination of the PDFs, so that the integral in Eq. (1) reduces to

24 $$\begin{aligned} T_I[f] = \int dx\, C_{I}(x) f(x). \end{aligned}$$

The details of the dataset and the definition of the flavour combination considered in this work are provided in Appendix A. Extensions of this analysis to theoretical predictions that are quadratic in the PDFs, multiple flavour combinations, alternative minimizers, and cross-validation tools are left for future work.

Finally, we emphasise that the results in this section, while obtained having in mind neural networks, apply to any fixed parametrization, including fixed functional forms [5–7] or kernels [28].

### Training in functional space

For analytical tractability, GD is described as a continuous flow of the parameters $\theta _{\mu }$ in training time *t* along the negative gradient of the loss function $\mathcal {L}$. Here the index $\mu$ runs over weights and biases in all layers of the network. For sufficiently small learning rates $\eta$, this continuous flow approximates the discrete GD trajectory in parameter space, as extensively discussed in Ref. [29]. The continuous gradient flow (GF) is then given by

25 $$\begin{aligned} \frac{d}{dt}&\theta _{t,\mu } = -\partial _\mu \mathcal {L}_t\, , \end{aligned}$$

where $\theta _{t,\mu }$ and $\mathcal {L}_t$ identify respectively the parameters and the loss function at training time *t*. In Eq. (25), $\partial _\mu \equiv \partial /\partial \theta _\mu$ denotes the partial derivative with respect to the single parameter $\theta _\mu$. The equation is therefore a scalar relation that holds component-wise for each $\mu$. We distinguish between the continuous training time *t* and the discrete epochs of GD, the latter denoted using the capital letter *T*. The two are related through the learning rate, $t = \eta T$ with $\eta = 10^{-5}$, and in the following we will use them interchangeably.

We focus here on quadratic loss functions that are obtained as the negative logarithm of Gaussian data distributions around their theoretical predictions,

26 $$\begin{aligned} \mathcal {L}_t = \frac{1}{2} \left( Y - T[f_t]\right) ^T C_Y^{-1} \left( Y - T[f_t]\right) \, , \end{aligned}$$

where $f_t$ is the output of the network at training time *t*, obtained from the time-dependence of the internal parameters. Here $C_Y$ is the covariance of the data, which includes statistical and systematic errors given by the experiments and also any theoretical error (e.g., missing higher orders in the theoretical predictions). Indices that are summed over are suppressed to improve the clarity of the equations. Note that the loss function at training time *t* is computed using the theoretical prediction $T[f_t]$, i.e., the result of Eq. (1) computed using the fields at training time *t*. For a quadratic loss, the gradient is

27 $$\begin{aligned} \partial _\mu \mathcal {L}_t = - \left( \partial _\mu f_t\right) ^T \left( \frac{\partial T}{\partial f}\right) _t C_Y^{-1} \epsilon _t\, , \end{aligned}$$

where, writing explicitly the data index,

28 $$\begin{aligned} \epsilon _{t,I} = Y_I - T_I[f_t]\, , \quad I=1, \ldots , N_{\textrm{dat}}\, . \end{aligned}$$

For the specific case of a quadratic loss function, the gradient is proportional to $\epsilon _t$, which is the difference between the theoretical prediction and the data at training time *t*. If at some point during the training the theoretical predictions reproduce all the data, the training process ends.

A further simplification is obtained in the case of data that depend linearly on the unknown function *f*. In the specific case of NNPDF fits, the integrals in Eq. (1) are approximated by a Riemann sum over the grid of *x* points,

29 $$\begin{aligned} T_I[f] \approx \sum _{i=1}^{N_{f}}\sum _{\alpha =1}^{N_{\textrm{grid}}} (\textrm{FK})_{Ii\alpha } f_{i\alpha }\, , \end{aligned}$$

and hence

30 $$\begin{aligned} \left( \frac{\partial T_I}{\partial f_{i\alpha }}\right) _t = (\textrm{FK})_{Ii\alpha }\, , \end{aligned}$$

which is independent of *t*. A few algebraic steps allow the flow of parameters $\theta$ to be translated into a flow for the fields,

31 $$\begin{aligned} \frac{d}{dt}&f_{t,i_1\alpha _1} = (\partial _\mu f_{t,i_1\alpha _1}) \frac{d}{dt}\theta _\mu = \Theta _{t,i_1\alpha _1i_2\alpha _2} (\textrm{FK})^T_{i_2\alpha _2I} \left( C_Y^{-1}\right) _{IJ} \epsilon _{t,J}\, , \end{aligned}$$

where we have defined the Neural Tangent Kernel [17]

32 $$\begin{aligned} \Theta _{t,i_1\alpha _1i_2\alpha _2} = \sum _\mu \partial _\mu f_{t,i_1\alpha _1} \partial _\mu f_{t,i_2\alpha _2}\, . \end{aligned}$$

As it will become clearer later, the NTK provides a powerful framework for understanding neural network dynamics during training. Originally developed by Jacot et al. [17] to analyse infinite-width feed-forward networks, the NTK theory has since been extended to diverse architectures including convolutional networks [30] and recurrent networks [31]. This theoretical framework has proven invaluable for characterizing learning dynamics and generalization properties across various network designs. We will see in the following and in Sect. 4 how the NTK can also provide useful insights in the context of PDF fitting.

In order to facilitate the discussion in Sect. 4.1, Eq. (31) can be rewritten in a more compact form. We first omit the indices and write, for instance,

33 $$\begin{aligned} \left( \frac{\partial T}{\partial f}\right) _t = (\textrm{FK})\, , \quad \Theta _t = \left( \partial _\mu f_t\right) \left( \partial _\mu f_t\right) ^T\, . \end{aligned}$$

Then, using the definition of the error in Eq. (28), we can rewrite Eq. (31) as

34 $$\begin{aligned} \frac{d}{dt}f_t = -\Theta _t M f_t + b_t\, , \end{aligned}$$

where

35 $$\begin{aligned} M&= (\textrm{FK})^TC_Y^{-1} (\textrm{FK})\, , \quad b_t = \Theta _t (\textrm{FK})^TC_Y^{-1} Y\, . \end{aligned}$$

Here *M* is a positive-semidefinite matrix that depends only on the data covariance and the FK tables that enter the theoretical predictions, while *b* is a vector that depends (amongst other quantities) on the central value of the data. We comment further on the role of *M* in Sect. 3.3. Note that any vector *f* that is in the kernel of $(\textrm{FK})$ is necessarily in the kernel of *M*, $\ker M$. In turn, the vectors in $\ker M$ do not contribute to the flow evolution, as seen explictly in Eq. (34).

Before moving to the next subsection, a few comments are in order. First, although derived in the context of neural networks, these equations do not refer to a specific parametrization. Indeed, these remain valid even when an explicit functional form is chosen to parametrize the PDFs, as in Refs. [5, 6, 28]. Second, it is interesting to observe that the flow equation, Eq. (34), depends on two matrices, $\Theta$ and *M*. The former encodes the model dependence, while the latter contains the physical information. The interplay between these two matrices is crucial for understanding the training dynamics, as discussed in Sect. 3.2.3. Finally, the NTK derived in Eq. (32) is inherently time-dependent in a complex way, which precludes any attempt at integrating Eq. (34) analytically. We come back to this point in Sect. 4.1, after discussing the properties of the NTK during training.

### Inside the training dynamics: an NTK perspective

From Eqs. (32) and (34), we observe that the NTK encodes the dependence on the architecture of the network and governs its training dynamics. The analysis of the NTK properties is thus crucial for understanding the behaviour of the network during training. We first discuss the properties of the NTK at initialisation, before moving to the training phase, where we provide a detailed study of the NTK in the context of the NNPDF methodology.

#### NTK at initialisation

Before training, the NTK is blind to data and depends on the *x*-grid of input and on the architecture, as shown in Eq. (32). The NTK is a function of the fields *f*, which are stochastic variables described by their joint probability distribution as discussed in Sect. 2. Therefore the NTK is also a stochastic variable, with its own probability distribution, which we represent as usual as a set of replicas.

**Fig. 6:**
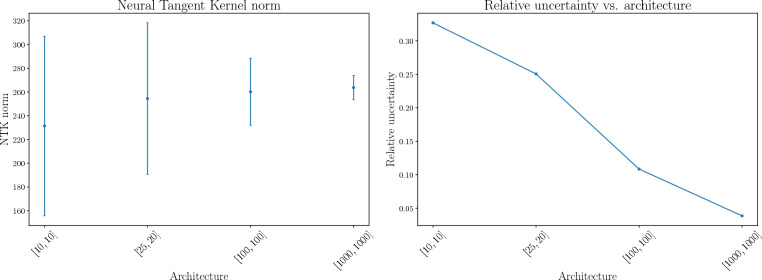
Frobenius norm of the NTK at initialisation, $\Vert \Theta _0 \Vert$, as a function of the width of the network. On the left, the central values and uncertainty bands are obtained as the mean and one-sigma deviation of the ensemble of networks. The plot on the right shows the relative uncertainty. It is interesting to note the decrease of the relative uncertainty as the architecture of the network is increased. For larger networks, the sensitivity to a change of the network parameters fluctuates less

It is argued in the literature that, in the large-width limit, the variance of the NTK over the set of replicas tends to zero with the width of the hidden layers (see, e.g., [20, 24]). In order to quantify the variation of the NTK, we start by computing the Frobenius norm of the NTK over an ensemble of networks for different architectures. For each architecture, we consider the standard deviation of the norm as a statistical estimator of the variations of the NTK. The result is displayed in Fig. 6. Even though the Frobenius norm is a coarse indicator of the variations of the NTK, the figure shows clearly that the variance of the norm becomes smaller with the size of the network, which is consistent with the theoretical expectation that the NTK should not fluctuate for infinite-width networks.^7^

**Fig. 7:**
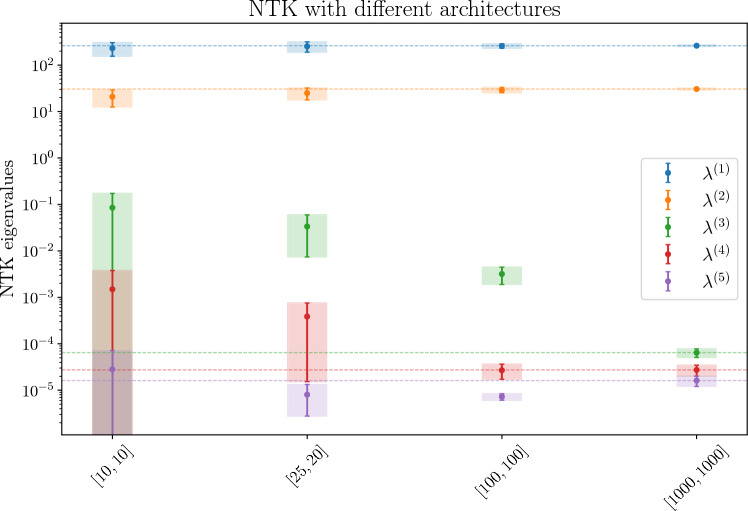
Spectrum of the NTK at initialisation for the architectures shown in Fig. 6. Error bands correspond to one-sigma uncertainties over the ensemble of networks. The hierarchy of the eigenvalues is independent of the size of the network. In agreement with the data in Fig. 6, the fluctuations of the eigenvalues decrease as the width of the layers is increased

A more quantitative description of the NTK at initialisation is provided by its spectrum, which is shown in Fig. 7 for four different architectures. Inspecting the figure, we see that the spectrum of the NTK is heavily hierarchical, and only few eigenvalues are actually non-zero.^8^ Such a hierarchy in the eigenvalues means that only a small subset of active directions can inform the network during training, as it will be discussed later. Note that, at least at initialisation, these observations do not depend on the architecture: the eigenvalues in Fig. 7 are mostly independent of the size of the network. Even though the logarithmic scale on the vertical axis may hide some small variations, it is clear that most eigenvalues remain constant within the error bars. On the other hand, the logarithmic scale emphasises that there are several orders of magnitude between eigenvalues for a given architecture; that hierarchical structure does not depend on the architecture. There is a downward fluctuation of the third eigenvalue for the largest architecture that we considered, but we do not have any evidence that this drop is a physical feature of the system, rather than a fluctuation. Finally, the variance of the set of eigenvalues over replicas decreases with increasing size, as expected.

#### NTK during training

We now discuss the behaviour of the NTK during training. To this end, we are going to adopt the so-called *closure tests* developed by the NNPDF collaboration. A closure test uses synthetic data, generated using a known set of PDFs, to train the neural network. The PDFs used for generating the data are called here *input* PDFs. The results of the training are then compared to the known input PDFs; the performance of the training algorithm and the NN architecture are assessed by quantifying the comparison between trained PDFs and input PDFs. Following the original presentation in Ref. [32], we distinguish three levels of closure tests, which are defined by the complexity of the data used to train the NNs. We use the standard NNPDF nomenclature and refer to these three levels as level-0 (L0), level-1 (L1), and level-2 (L2) closure tests, and we denote the input PDFs used to generate the data as $f^\textrm{in}$. The definitions of these three levels of data are given in Appendix A.

For each of the closure-test data given above, we perform a fit of the triplet combination $T_3$ using the simplified version of the NNPDF methodology that we discussed above. We initialise an ensemble of $N_\textrm{rep}= 100$ replicas with identical architecture, training each replica independently using GD optimization. Throughout the training process, we track the evolution of the NTK to understand how the network’s effective dynamics change as it learns the target function.

**Fig. 8:**
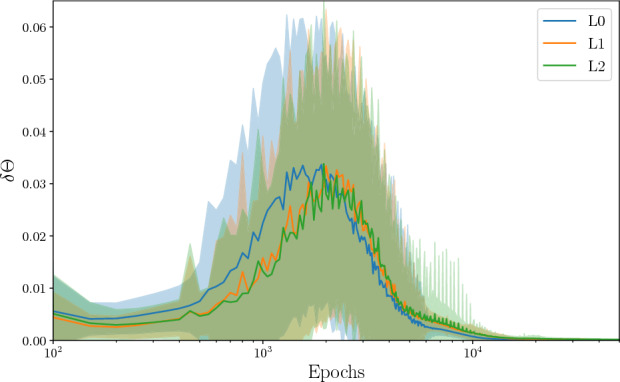
Relative variation of the NTK during training for L0, L1, and L2 data. Error bands correspond to one-sigma uncertainties over the ensemble of networks

**Onset of lazy training** As a first estimator of the variation of the NTK, we show in Fig. 8 the Frobenius norm of the variation during training, normalized by the Frobenius norm of the NTK itself,

36 $$\begin{aligned} \delta \Theta _t = \frac{\Vert \Theta _{t+1} - \Theta _t \Vert }{\Vert \Theta _t \Vert } , \end{aligned}$$

for the three different datasets, L0, L1, and L2. Inspecting the plot reveals that the NTK undergoes significant changes during the initial phase of training, with the relative variation $\delta \Theta _t$ reaching values as high as $6\%$. This indicates that our settings differ from the standard picture of lazy training in the context of very wide networks, as discussed, e.g., in Refs. [17, 19, 20], where the NTK is expected to be independent of the flow time *t*. Remarkably, we do not observe a dependence on how data have been generated, indicating that the NTK dynamics is basically unaffected by the noise that affects the data.

After this initial phase – corresponding approximately to the first 20,000 epochs in our experiment – the NTK tends to stabilize. These two regions will be referred to as the *rich* and *lazy* training regimes, respectively, in keeping with the standard terminology adopted in the literature (see, e.g., Ref. [33] where two similar regimes were also identified). We do not comment any further on the implications of the lazy regime, and postpone the discussion to Sect. 4.1.

**Fig. 9:**
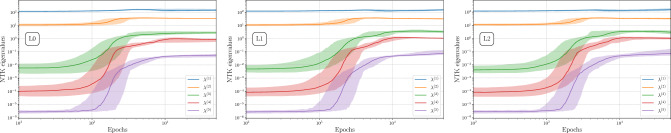
Evolution during training of the first five eigenvalues of the NTK using L0 (left), L1 (center), and L2 (right) data. Solid lines represent the median over the ensemble of networks, while solid bands correspond to 68% confidence level. Note that the subdominant eigenvalues $\lambda ^{(3)}$, $\lambda ^{(4)}$ and $\lambda ^{(5)}$ have increased by one or two orders of magnitude by the end of the rich training phase

**Eigenvalues during training** Further insight on the evolution of the NTK can be obtained by studying its eigensystem as a function of the training time. In Fig. 9 we report the variation of the first five eigenvalues of the NTK, using the standard NNPDF architecture, for L0, L1, and L2 data. We see that the hierarchical structure observed at initialisation is preserved, but the size of the subdominant eigenvalues increases significantly in the early stages of training – by one or two orders of magnitude depending on the specific eigenvalue.

**Fig. 10:**
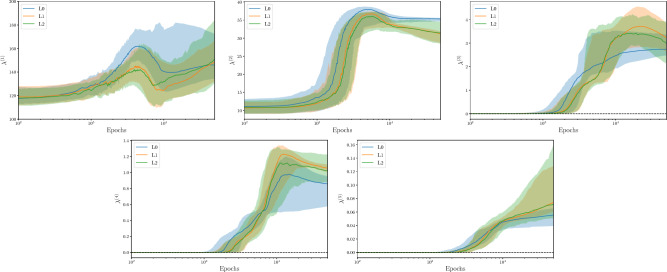
The first five eigenvalues of the NTK for L0, L1, and L2 data. Solid lines represent the median over the ensemble of networks, while solid bands correspond to 68% confidence level. Each plot corresponds to a different eigenvalue, as indicated by the label on the vertical axis. Note the different scales on the vertical axes, which reflects the hierarchy of eigenvalues discussed above. Different colours correspond to different synthetic data, the agreement between these bands confirms that the evolution of the eigensystem of the NTK does not depend on the level of noise in the data

In Fig. 10, the same first five eigenvalues of the NTK are displayed for L0, L1, and L2 data. We observe a common pattern across all data types, consistently with the observation made before in Fig. 8. This indicates the NTK evolution is insensitive to the noise included in the synthetic data. The increase of the subdominant eigenvalues, combined with the analysis of Eqs. (55) and (56) in Sect. 4, suggests that more “physical” features become learnable before lazy training sets in.

**Fig. 11:**
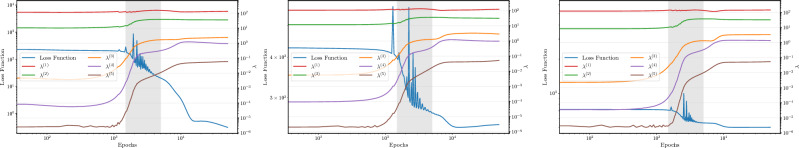
Variation of the loss function overlaid with the first five eigenvalues for a selected replica over the ensemble using L0 (left), L1 (center), and L2 (right) data. Left scale refers to the loss, while the right scale refers to the eigenvalues

**Connection with the loss function** Finally, in Fig. 11 we show the variation of the loss function during training, overlaid with the first five eigenvalues of the NTK, for a selected replica over the ensemble. It is interesting to see that in correspondence with the sudden variation of the subdominant eigenvalues, the loss function drops significantly, at the cost of an instability localised in the descent. We interpret this as the network learning new features, changing its internal representation to accommodate the new information. After this initial phase, the eigenvalues stabilize and the loss function decreases smoothly, as expected in the lazy training regime.

As it will be extensively discussed later in Sect. 4, the eigenvalues and eigenvectors of the NTK play a special role. Indeed, the output *f* can be decomposed into the basis of eigenvectors of the NTK. Hence the eigenvectors corresponding to the larger eigenvalues can be interpreted as *learnable* features, while the small (or zero) eigenvalues correspond to directions in which the field *f* never evolves during training.

**Fig. 12:**
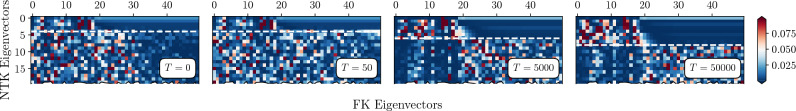
Matrix *A* as defined in Eq. (37) for L2 data and for a single replica of the NTK. The matrix is shown at different epochs of the training process, indicated in the top of each panel. The white dashed line indicates the cut-off tolerance that we impose on the eigenvalues of the NTK (see Appendix C)

#### Eigenvectors and alignment of the NTK

It has been argued above that there is a non-trivial interplay between the eigenspace of the NTK and that of the matrix *M*. Indeed, the former encodes the model dependence, while the latter yields physical information. Of course the two matrices are independent at initialisation, and we do not expect any alignment pattern between the two. However, this picture does change during training, as the NTK evolves and the model learns the target function. To quantify this alignment, we define the matrix *A*,

37 $$\begin{aligned} A_{kk'} = \left( z^{(k)}, v^{(k')} \right) ^2 = \cos ^2(\theta _{kk'}) , \end{aligned}$$

where $z^{(k)}$ and $v^{(k')}$ are the *k*-th and $k'$-th eigenvectors of the NTK and *M*, respectively. The matrix *A* is thus a measure of the alignment between the eigenspaces of the two matrices. The rows of the matrix correspond to the eigenvectors of the NTK, ordered by the value of the corresponding eigenvalues, with the eigenvectors corresponding to the larger eigenvalues at the top of the matrix. The columns correspond to eigenvectors of the matrix *M*, also ordered by the values of the corresponding eigenvalues, with the largest eigenvalues to the left in this case. In Fig. 12, we show the matrix *A* at different epochs of the training for L2 data and a single NTK replica.

The blue rectangle in the top right corner of the matrix shows that the eigenvectors of the NTK corresponding to the largest eigenvalues are orthogonal to the eigenvectors of *M* that are in the kernel of *M*, i.e., the directions that do not contribute to the observables. It is useful to remember that the largest eigenvalues of the NTK correspond to the directions that are orthogonal to $\ker \Theta$, i.e., the directions that are learnable during the training process. In order to have a robust training process, we expect these learnable directions to align with the directions that actually contribute to the loss functions, which are the ones corresponding to the largest eigenvalues of *M*. Consistently with this intuition, we see that the size of this blue rectangle increases with training time. In particular, it is clear from our plot that it becomes deeper by the onset of the lazy training regime: more of the learnable directions – the *features* that the network can learn – are aligned with the directions that contribute most to the observables.

**Fig. 13:**
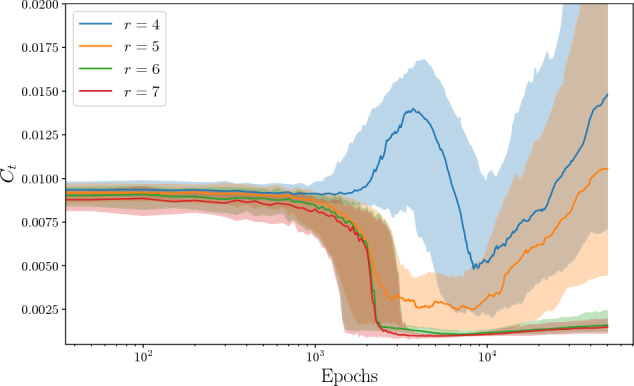
Reconstruction error $C_t(r)$ as defined in Eq. (38) as a function of the training time and for different numbers of eigenvectors *r*. Note that $f^\textrm{in}$ lies entirely in the subspace spanned by the first four eigenvectors of the NTK by the onset of the lazy training regime. We see that the NTK has aligned its features with the physically relevant directions of the problem

We remark that the eigenvectors of the NTK form an orthonormal basis in $\mathbb {R}^{N_{\textrm{grid}}}$ at any epoch of the training process. We have also shown that only a subset of these eigenvectors contribute to the training dynamics. We may thus wonder how expressive the vectors belonging to the subspace orthogonal to the kernel are, i.e., how well the internal representation of the neural network can reconstruct the input function $f^\textrm{in}$ used to generate the data. In order to quantify this aspect, we define a new figure of merit

38 $$\begin{aligned} C_t(r) = 1 - \sum _{k=1}^{r} \frac{(z^{(k)}, f^\textrm{in})^2}{\Vert f^\textrm{in}\Vert ^2} , \end{aligned}$$

which measures the reconstruction error of the input function $f^\textrm{in}$ when projected onto the subspace spanned by the first *r* eigenvectors of the NTK at training time *t*. We show $C_t(r)$ as a function of the training time and for different choices of *r* in Fig. 13. Inspecting the figure, we see that in the early stages of training the reconstruction error does not change significantly with time. This behaviour is shared across all values of *r*. In fact, in this first phase the NTK has not yet found a suitable internal representation and the inclusion of more eigenvectors corresponding to yet undiscovered directions – those associated with a small eigenvalue as in Fig. 9 – does not result in an improvement of the reconstruction of $f^\textrm{in}$. Conversely, once the onset of lazy training is approached, we identify two distinct behaviours depending on the number of used eigenvectors. If we include the eigenvectors up to the modes discovered during training (e.g., $r=6$ and $r=7$ in the figure), the reconstruction error drops significantly and remains almost constant throughout the rest of the learning process. On the other hand, if we consider fewer eigenvectors (e.g., $r=4$ and $r=5$ in the figure), the reconstruction error becomes larger and grows indefinitely with training time. This results show us that the subset of eigenvectors orthogonal to $\ker \Theta$ is capable of providing an effective lower dimensional basis, provided that the new modes discovered by the NTK during training are included. Note that the reconstruction of the input function improves as long as the included eigenvectors do not belong to $\ker \Theta$ – being noise, they would not bring any physical information.

**Fig. 14:**
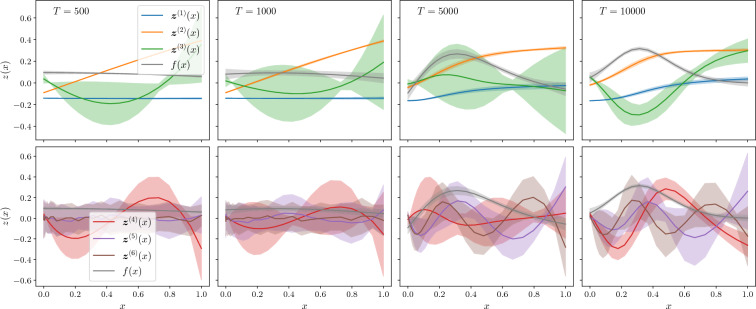
First five eigenvectors of the NTK at different training times and as function of the input *x*-grid. We also show the output of the network at the same training time, which is displayed in gray. L1 data is used

A complementary picture is displayed in Fig. 14. Here, we show the eigenvectors of the NTK at different training times as functions of the *x*-grid, denoted by $z^{(i)}$. Together with the eigenvectors, we also show the output of the trained neural network at the corresponding training time. From these plots, we see that as the training progresses, the shape of the eigenvectors becomes more structured in order to reproduce the output function. Again, this conclusion supports the observations made previously on various occasions, that during training the neural network is changing its internal representation and the NTK encodes this information.

### Regularisation of the inverse problem and the role of the NTK

The discussion above characterised the training dynamics of the network in terms of the NTK. It did not, however, address how the combination of a parametrisation and an optimisation process can solve the ill-defined inverse problem of PDF fitting. Before concluding this section, we now discuss two complementary perspectives that expose the ill-defined nature of the inverse problem and the role of the parametrisation in regularising it. The discussion also introduces the eigenspace decomposition that is used extensively in Sect. 4.

**Fig. 15:**
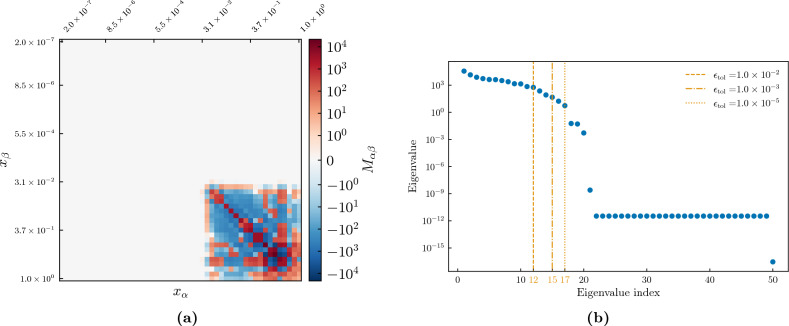
**a** Entries of the operator *M* for the BCDMS dataset. **b** Singular values of the operator *M*, shown in logarithmic scale. The three vertical dashed lines correspond to the three choices of $\epsilon _\textrm{tol}$ discussed in the main text. The points crossed by these lines are included in the pseudo-inverse

**Fig. 16:**
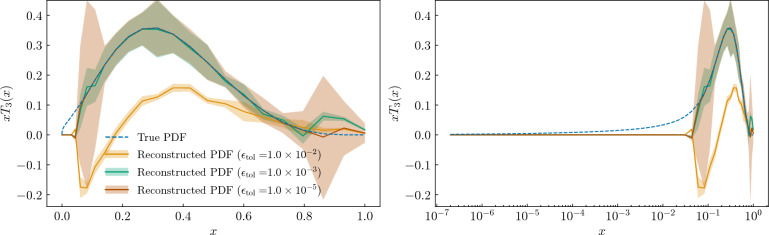
Solution of the inverse problem as in Eq. (40) with the relative thresholds as in Fig. 15b. The dashed line indicates the underlying law used to generate the artificial data

**The inverse problem in functional space.** One could attempt to determine *f* by directly minimising the loss in Eq. (26) in functional space. Taking the derivative with respect to *f* and setting it to zero yields the stationary condition

39 $$\begin{aligned} M f = (\textrm{FK})^T C_Y^{-1} Y\,, \end{aligned}$$

with *M* defined as in Eq. (35). Here the time dependence is dropped, as no training process is involved. The transpose $(\textrm{FK})^T$ acts as the *adjoint* of the forward map $(\textrm{FK})$: it takes a vector in data space, weighted by the data uncertainties, and returns the linear combination in PDF space that the data probe. Crucially, $(\textrm{FK})^T$ is not the inverse of $(\textrm{FK})$. Its range is the orthogonal complement of $\ker (\textrm{FK})$, so it carries no information about PDF directions in $\ker (\textrm{FK})$ – precisely the directions to which the data are insensitive. The composite operator $M=(\textrm{FK})^T C_Y^{-1} (\textrm{FK})$ inherits this blind spot, with $\ker M= \ker (\textrm{FK})$.^9^ The solution of Eq. (39) is therefore not unique. It decomposes as $f = f_{\ker (M)}+ f_{\textrm{Im}(M)}$, where $f_{\ker (M)}\in \ker M$ is undetermined by the data and the particular solution in $\text {Im}\, M$ is

40 $$\begin{aligned} f_{\textrm{Im}(M)}= M^+(\textrm{FK})^T C_{Y}^{-1} Y, \end{aligned}$$

with $M^+$ the Moore–Penrose pseudo-inverse of *M*. The pseudo-inverse acts as a deconvolution operator on $\text {Im}\, M$, and acquires a statistical interpretation: assuming $Y\sim \mathcal {N}(\bar{Y}, C_Y)$, we have

41 $$\begin{aligned} \begin{aligned}&\mathbb {E}[f_{\textrm{Im}(M)}] = M^+ (\textrm{FK})^T C_{Y}^{-1} \bar{Y},\\&\text {Cov}[f_{\textrm{Im}(M)}, f_{\textrm{Im}(M)}^T] = M^+ M M^+ = M^+, \end{aligned} \end{aligned}$$

which shows that $M^+$ is itself the covariance of the reconstructed solution in $\text {Im}\, M$. In practice, the spectrum of *M* for realistic datasets is highly hierarchical and only a small subspace is numerically accessible. Figure 15a displays *M* for the BCDMS dataset used in this work, while Fig. 15b shows its singular values: many directions are essentially null and the non-zero eigenvalues span several orders of magnitude. The effective rank of *M* is therefore set by a relative threshold $\epsilon _\textrm{tol} \times \max (s)$, the choice of which is arbitrary and propagates to both central values and uncertainties of the reconstructed solution. As shown in Fig. 16, a tight threshold ($\epsilon _\textrm{tol}=10^{-2}$) excludes too many modes and underfits the underlying law; a loose one ($\epsilon _\textrm{tol}=10^{-5}$) recovers it but with unstable uncertainties at the boundary; intermediate values are a compromise but still leave uncertainties unstable across the *x* range. On top of the arbitrariness of $\epsilon _\textrm{tol}$, the solution provides no robust estimate in the extrapolation region (where any $f_{\ker (M)}\in \ker (\textrm{FK})$ can be added) and is not constrained to be smooth across the boundary between the constrained and unconstrained regions. These pitfalls motivate the use of more robust methods, such as the one studied in this work.

**Regularisation through a linear parametrisation** A simple example can help illustrate the role of the parametrisation in regularising the inverse problem. Consider a function linear in the parameters,

42 $$\begin{aligned} f(x;\theta ) = \sum _{i=1}^{n} \theta _i \phi _i(x), \end{aligned}$$

with fixed basis functions $\phi _i(x)$ (e.g., polynomials).^10^ Evaluated on the *x*-grid, the function reads

43 $$\begin{aligned} f = \Phi \theta , \end{aligned}$$

where $\Phi _{\alpha i} \equiv \phi _i(x_{\alpha })$ is the Jacobian of *f* with respect to $\theta$, evaluated on the *x*-grid with $\alpha = 1, \dots , N_{\textrm{grid}}$. Here we are using the same compact and index-less notation introduced in Sect. 3.1. The minimum of the loss in Eq. (26) with respect to the parameters $\theta$ satisfies

44 $$\begin{aligned} \Phi ^T (\textrm{FK})^T C_Y^{-1} \left( Y - (\textrm{FK})f(\tilde{\theta })\right) = 0\,, \end{aligned}$$

where $\tilde{\theta }$ denotes the vector of optimal parameters. By observing that, for the linear model in Eq. (43), the NTK reduces to the time-independent matrix $\Theta = \Phi \Phi ^T$, we can multiply both sides of the previous equation by $\Phi$,

45 $$\begin{aligned} \Theta M f(\tilde{\theta }) = \Theta (\textrm{FK})^T C_Y^{-1} Y, \end{aligned}$$

which recasts the stationary condition in terms of the NTK, similar to the flow equation in Eq. (34). In general, $\Theta$ is only positive *semi*-definite, with a non-trivial kernel whenever $n < N_{\textrm{grid}}$ – as illustrated by the spectrum at initialisation in Fig. 7. The eigenvalue equation,

46 $$\begin{aligned} \Theta z^{(k)} = \lambda ^{(k)} z^{(k)}, \end{aligned}$$

provides a basis of $\mathbb {R}^{N_{\textrm{grid}}}$ separating the kernel of the NTK from its image. The kernel collects the directions in functional space that the parametrisation cannot reach, irrespective of the data. We introduce the notation

47 $$\begin{aligned}&f^\parallel _{k} = \left( z^{(k)}, f\right) \, , \quad \text {if}\ \lambda ^{(k)} = 0\, , \end{aligned}$$

48 $$\begin{aligned}&f^\perp _{k} = \frac{1}{\sqrt{\lambda ^{(k)}}} \left( z^{(k)}, f\right) \, , \quad \text {if}\ \lambda ^{(k)} \ne 0\, , \end{aligned}$$

with the scalar product defined as $\left( f'_{t'}, f_t\right) = \sum _{i,\alpha } f'_{t',i\alpha } f_{t,i\alpha }$. In this linear case, $f = \Phi \theta$ lies by construction in $\textrm{im}\,\Phi \equiv \textrm{im}\, \Theta$ for *any* choice of the parameters $\theta$. Hence, the component $f^{\parallel } = 0$ identically: the parametrisation forces the solution to be orthogonal to the kernel of the NTK. Projecting Eq. (45) onto $z^{(k)}$ for $\lambda ^{(k)} \ne 0$, and denoting by $d_\perp$ the dimension of the subspace orthogonal to $\text {ker}\ \Theta$, yields

49 $$\begin{aligned} \sum _{k'}^{d_\perp } H_{kk'}^{\perp } f_{k'}^{\perp } = \sqrt{\lambda ^{(k)}} \left( z^{(k)}, (\textrm{FK})^T C_{Y}^{-1} Y \right) , \end{aligned}$$

where we have introduced the $d_\perp \times d_\perp$ symmetric matrix

50 $$\begin{aligned} H^\perp _{kk'} = \sqrt{\lambda ^{(k)}} \left( z^{(k)}, M z^{(k')}\right) \sqrt{\lambda ^{(k')}}. \end{aligned}$$

We refer to $H^\perp$ as the *flow* (or *training*) *Hamiltonian* for reasons that become clear in the next section: training takes place in the subspace orthogonal to $\ker \Theta$. Its dynamics is set by an interplay of the architecture (encoded in the NTK) and the data (encoded in *M*), thus dictating the dynamics of the training process. The crucial observation is that $H^\perp$ is invertible whenever $\ker M \cap \text {Im}\,\Theta = \{0\}$. Outside pathological choices of basis $\{\phi _i\}$ – where the parametrisation is characterised by non-zero directions $z^{(k)}$ such that $M z^{(k)} = 0$ for some $\lambda ^{(k)} \ne 0$ – this condition is satisfied. The projected system is then solved unambiguously,

51 $$\begin{aligned} f_k^{\perp } = \sum _{k'}^{d_\perp } \left( H^\perp \right) ^{-1}_{kk'} \sqrt{\lambda ^{(k')}} \left( z^{(k')},\, (\textrm{FK})^T C_{Y}^{-1} Y\right) , \end{aligned}$$

and the full solution is reconstructed as

52 $$\begin{aligned} f = \sum _{k}^{d_\perp } \sqrt{\lambda ^{(k)}}\, f_k^{\perp }\, z^{(k)}\,. \end{aligned}$$

Comparing with Eq. (40), the role of the parametrisation is now made explicit: the singular operator *M* has been replaced by its projection $H^\perp$ onto the image of the NTK, which is non-singular under mild assumptions. The parametrisation thus regularises the inverse problem by restricting the solution to the subspace of functions it can represent *and* where the data-induced metric is non-degenerate.

**From the linear analogue to the NN case.** The linear model captures the essential mechanism by which a parametrisation regularises the inverse problem, but it differs from the NN case in two important respects. First, in a NN the NTK $\Theta _t$ depends on the training time *t*, so the decomposition in Eqs. (47) and (48) is itself time-dependent, and modes can transfer between $\ker \Theta _t$ and $\text {Im}\, \Theta _t$ during the rich phase. Second, when the parametrisation populates $\ker \Theta _t$, the parallel component $f_t^{\parallel }$ no longer vanishes: in the lazy regime, where the NTK becomes approximately constant, gradient descent has no gradient along $\ker \Theta$, and $f_t^{\parallel }$ is frozen at the value $f_0^{\parallel }$ inherited from the onset of the lazy training. These unconstrained directions are not pinned down by the loss but by the initial condition, and the randomness propagates from the initialisation into the trained solution. We make this picture quantitative in Sect. 4, deriving a closed-form expression for $f_t$ in the lazy regime using the same eigenspace decomposition introduced here.

## Lazy training in NNPDF

In the previous section we observed that the NTK is able to capture the main features of the training process, and that its time evolution is characterised by a rapid initial transient, followed by a slower evolution during the rest of the training. We also showed, by means of a simplified analogy, the role of the parametrisation in regularising the ill-defined inverse problem. We now turn our attention to this last stage of the training, where the NTK has stabilised and becomes approximately constant. In doing so, we build upon the results presented in Refs. [17, 19] and extend them to the case of NNPDF. In the following, we derive the analytical solution of the flow equation, which allows us to write an explicit expression for the trained field as a function of the field at initialisation and the data.

### Analytical results

The lazy training regime is characterised by a slow-evolving NTK. We denote as $t_\textrm{ref}$ the time at which the onset of this regime occurs. The NTK is then *frozen* to its value at $t_\textrm{ref}$, and from this time onward the NTK is taken to be constant

53 $$\begin{aligned} \Theta _t = \Theta _{t_\textrm{ref}} \equiv \Theta , \quad \text {for } t \ge t_\textrm{ref}, \end{aligned}$$

and we use the same convention as in Sect. 3 to distinguish between the continuous time $t_\textrm{ref}$ and the discrete epoch $T_\textrm{ref}$. The flow equation can then be written as

54 $$\begin{aligned} \frac{d}{dt}f_t = -\Theta M f_t + b\, , \end{aligned}$$

where *M* and *b* are defined as in Eq. (35). Note that now neither $\Theta$ nor *b* depend on the training time *t*. We solve this first-order linear differential equation by projecting $f_t$ onto the basis spanned by the eigenvectors of the NTK, defined in Eq. (46). Specifically, we distinguish the two components $f^\parallel _{t,k}$ and $f^\perp _{t,k}$ introduced in Eqs. (47) and (48), now supplemented with explicit time dependence.

**Fig. 17:**
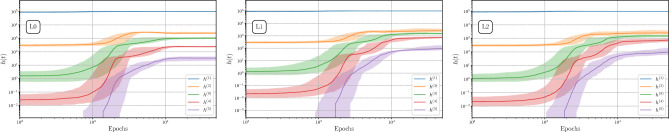
Evolution during training of the first five eigenvalues of $H^{\perp }$ using L0 (left), L1 (center), and L2 (right) data. Solid lines represent the median over the ensemble of networks, while solid bands correspond to 68% confidence level. Note that the subdominant eigenvalues $\lambda ^{(3)}$, $\lambda ^{(4)}$ and $\lambda ^{(5)}$ have increased by one or two orders of magnitude by the end of the rich training phase

**Fig. 18:**
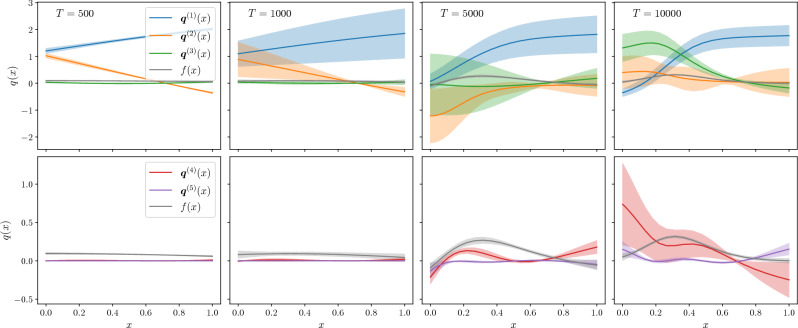
First five eigenvectors of $H^{\perp }$ projected onto PDF space, denoted as $q^{(i)}$, for different training times and as a function of the input *x*-grid. We also show the output of the network at the same training time, which is displayed in grey. L1 data is used

One can readily see that the components in the kernel of $\Theta$, $\text {ker}\ \Theta$, do not evolve during training,^11^

55 $$\begin{aligned} \frac{d}{dt}f^\parallel _{t,k} = 0 \quad \Longrightarrow \quad f^\parallel _{t,k} = f^\parallel _{0,k}\, . \end{aligned}$$

This means that the final solution is affected by an irreducible noise that is purely dictated by the initial condition. The flow equation for the orthogonal components can be written as

56 $$\begin{aligned} \frac{d}{dt}f^\perp _{t,k} = - H^\perp _{kk'} f^\perp _{t,k'} + B^\perp _{k}\, , \end{aligned}$$

where $H^{\perp }$ is the *training Hamiltonian* defined in Eq. (50) and

57 $$\begin{aligned} B^\perp _k = -\sqrt{\lambda ^{(k)}} \left[ \sum _{k'}^{d_{\parallel }}\left( z^{(k)}, M z^{(k')}\right) f^\parallel _{0,k'} - \left( z^{(k)}, (\textrm{FK})^TC_Y^{-1} Y\right) \right] , \end{aligned}$$

where $d_\parallel$ denotes the dimension of the subspace $\text {ker}\ \Theta$. The indices on quantities that have a $\perp$ suffix only span the space orthogonal to the kernel of $\Theta$, while the indices on quantities that have a $\parallel$ suffix span the kernel. As discussed in Sect. 3.3, $H^{\perp }$ is positive definite under the mild assumption $\ker M \cap \text {im}\,\Theta = \{0\}$. Therefore, its eigenvalues and eigenvectors,

58 $$\begin{aligned} H^\perp _{kk'} w^{(i)}_{k'} = h^{(i)} w^{(i)}_{k}, \end{aligned}$$

can be used to solve the flow equation similar to the linear-in-parameters analogue of Sect. 3.3. In Fig. 17 we show the evolution during training of the first five eigenvalues of $H^{\perp }$ for the three different closure datasets. In Fig. 18 we show, for different training times, the first five eigenvectors of $H^{\perp }$ projected onto the PDF space according to

59 $$\begin{aligned} q_{\alpha }^{(i)} = \sum _{k=1}^{d_\perp } \sqrt{\lambda ^{(k)}} z^{(k)}_\alpha w^{(i)}_k, \end{aligned}$$

which follows from the notation introduced in Eq. (48). It should not come as too much of a surprise that the eigenvalues and eigenvectors of $H^{\perp }$ have a similar behaviour to those of the NTK (see Figs. 9 and 14), from which they are constructed. However, we see that the eigenvalues $h^{(i)}$ are are roughly three orders of magnitude larger than those of the NTK.

**Fig. 19:**
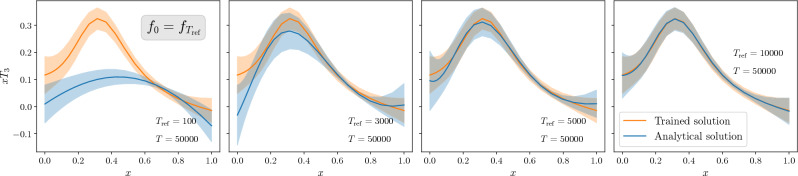
Comparison of the trained and analytical evolution at the end of training. Each panel corresponds to a different frozen NTK, whereby the analytical solution is computed starting from $f_{T_\textrm{ref}}$. The orange curve represents the final trained function after 50,000 iterations of GD, and is the same across panels. Error bands represent one-sigma uncertainties across replicas. L2 data is used

The solution to the flow equation, whose derivation is detailed in Appendix D, can be written as

60 $$\begin{aligned} f_{t,\alpha } = U(t)_{\alpha \alpha '} f_{0,\alpha '} + V(t)_{\alpha I} Y_{I}\,, \end{aligned}$$

where the evolution operators *U*(*t*) and *V*(*t*) have lengthy, yet explicit, expressions that we also report in Appendix D. Eq. (60) is the main result of this section. It shows that the training process can be described as the sum of a linear transformation of the initial fields $f_{0,\alpha }$, and a linear transformation of the data $Y_I$. The two transformations depend on the flow time *t* and are given by the evolution operators *U*(*t*) and *V*(*t*). Figure 19 compares the analytical solution with the trained function at the end of training, for different choices of the frozen NTK. The NN is trained using the numerical GD until $t_\textrm{ref}$, at which point the NTK is frozen. The evolution time *t* used in the analytical solution is the difference between the total training time and $t_\textrm{ref}$; the initial condition for the analytical solution is the trained solution at $t_\textrm{ref}$. Central value and uncertainty bands are obtained by computing the analytical solution for each replica of the initial condition and frozen NTK.^12^ As expected, the closer $t_\textrm{ref}$ is to the onset of the lazy regime, the better the agreement between the analytical solution and the trained function.

**Fig. 20:**
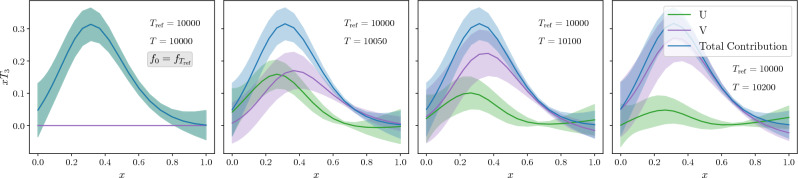
Decomposition of the analytical solution into the two contributions from *U* and *V* at different training times. The frozen NTK is fixed across panels, and corresponds to the one at $T_\textrm{ref} = 10000$. The initial condition for the analytical solution is always $f_{T_\textrm{ref}}$. As in Fig. 19, L2 data is used

A complementary perspective is provided in Fig. 20, where the analytical solution is decomposed into the two contributions from *U* and *V*. In each panel, the initial condition $f_{t_\textrm{ref}}$ is evolved analytically for different training times by keeping the frozen NTK fixed. We see that as training proceeds, the contribution from *U* is progressively suppressed, in accordance with the observation made above. On the other hand, the contribution from *V* grows and becomes dominant at later epochs, indicating that the trained function is mostly determined by the data, rather than the initial condition of the network. We also observe that such behaviour happens quite rapidly – in a training time interval $\Delta T \approx 200$ – since the time scales in the analytical solution are determined by the inverse of the eigenvalues of $H^\perp$, which are typically large.

The analytical solution in Eq. (60) sheds new light on the numerical training of a neural network. Given these results, it is natural to ask whether the information encoded in the NTK alone can drive training, independently of the initial condition, i.e., whether the analytical solution can be used to perform kernel learning. We address this question in the following section.

### Numerical results

The results shown in Sect. 4.1 and Sect. 3.2.2 support the idea that the NTK is capable to encode in its eigenvectors the physical features learned during training. We now probe this idea further by employing the analytical solution in Eq. (60) *à la* kernel learning, i.e., by applying it to an initial condition drawn from the prior distribution. In the following, we choose the initial condition to be an ensemble of networks at initialisation as in Fig. 5, whose architecture is the same as the one used in the training. This represents our prior assumption on the space of functions, which is then updated using the data and the NTK frozen at $t_\textrm{ref}$.

**Fig. 21:**
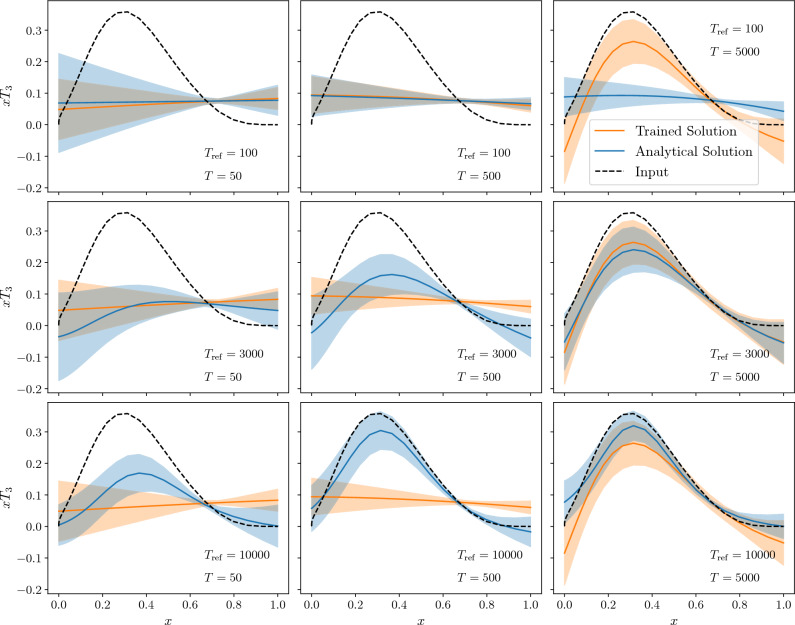
Comparison of the trained (orange) and analytical (blue) evolution starting from an ensemble of networks at initialisation as the initial condition. Each row corresponds to a different frozen NTK, while the columns represent different training times. The dashed line represents the input function used to generate the synthetic data, i.e., the *true* result. L2 data is used

**Fig. 22:**
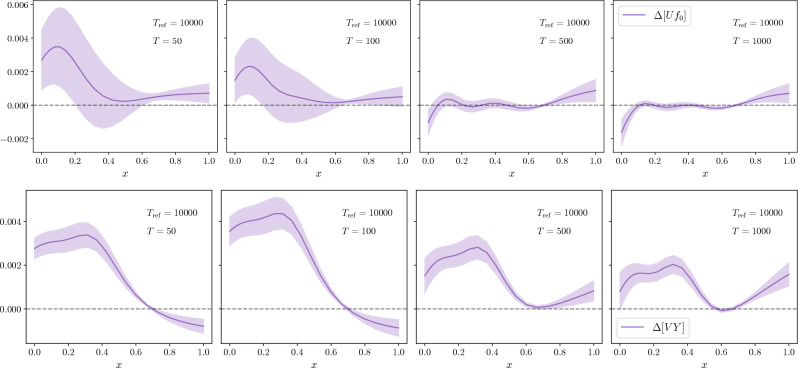
Behaviour of $\Delta [U(t)f_0]$ and $\Delta [V(t)Y]$, as defined in Eqs. (70) and (71), as functions of the training time. The operators *U*(*T*) and *V*(*T*) are constructed by taking the NTK at $T_\textrm{ref} = 10000$, which is fixed across panels. The uncertainties are extracted from the bootstrap ensemble as discussed in the text

**Fig. 23:**
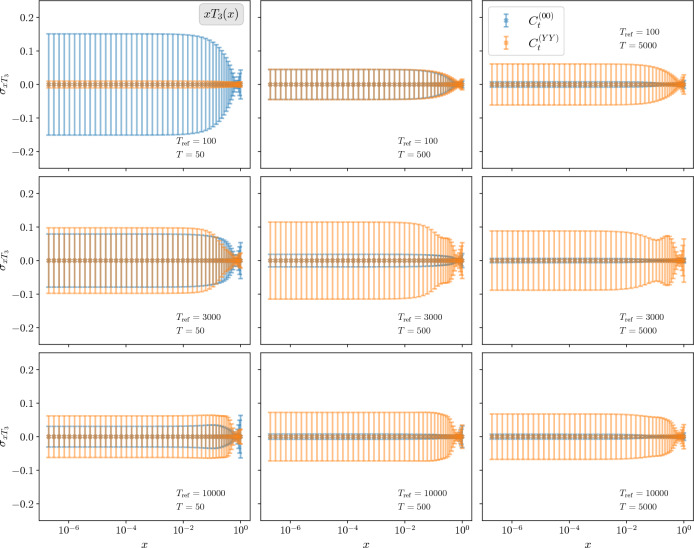
Decomposition of the error budget of the trained fields into the two components from the initial condition (blue) and from the data (orange), as defined in Eqs. (64) and (66). Each row corresponds to a different frozen NTK, while the columns represent different training epochs. L2 data is used. We see that if the NTK is taken at later stages of training, the contribution from the initial condition is severely suppressed towards the end of training

**Fig. 24:**
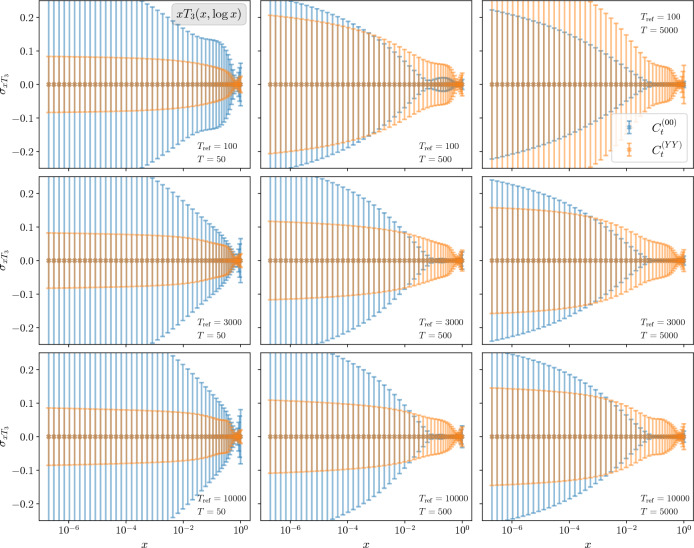
Similar to Fig. 23, but now for the case of scaled input $f(x, \log x)$

**Fig. 25:**
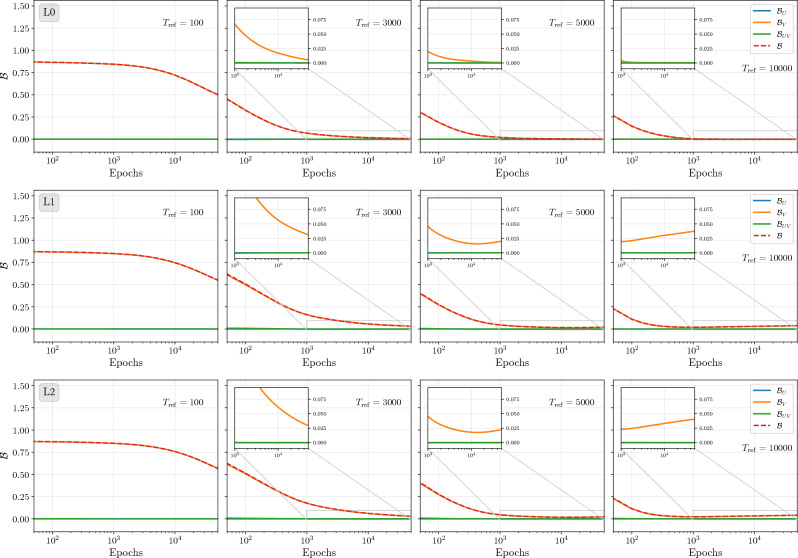
Bias decomposition as in Eq. (76) as a function of the training time for L0 (top), L1 (middle) and L2 (bottom) data. The curves are computed using the analytical result in Eq. (60). Each column corresponds to a different frozen NTK

**Fig. 26:**
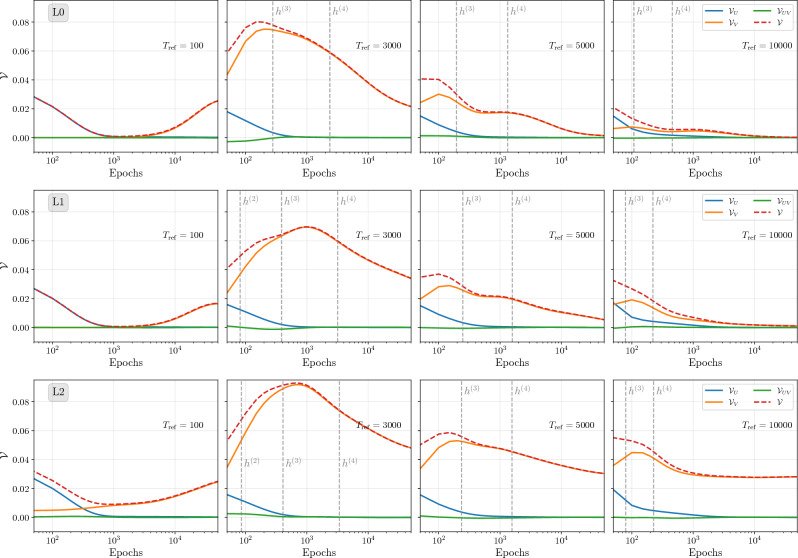
Variance decomposition as in Eq. (82) as a function of the training time for L0 (top), L1 (middle) and L2 (bottom) data. The curves are computed using the analytical result in Eq. (60). Each column corresponds to a different frozen NTK. The vertical dashed lines in grey indicate the inverse of the eigenvalues of $H^\perp$, $1/h^{(i)}$

**Fig. 27:**
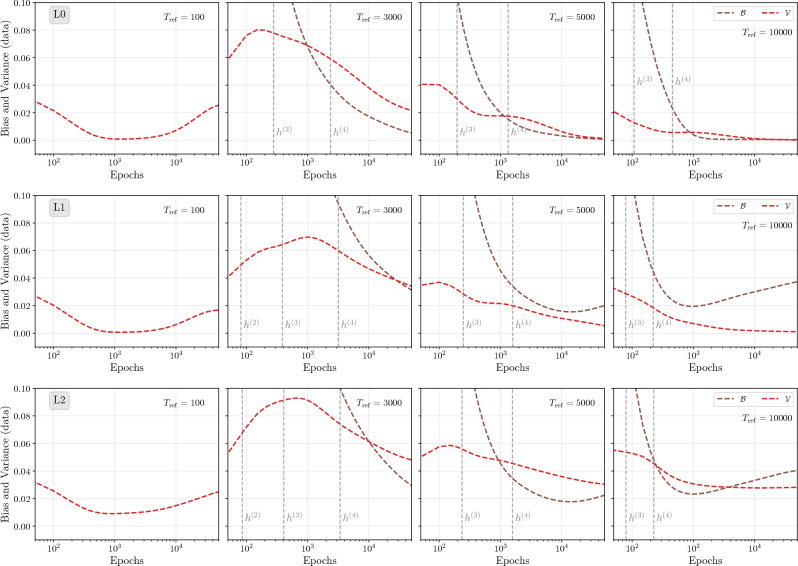
Comparison of the total contribution of bias and variance in the space of the data as a function of the training time for L0 (top), L1 (middle) and L2 (bottom) data. The curves are the same as in Figs. 25 and 26, displayed here together. Each column corresponds to a different frozen NTK. In first column, the bias is off-scale. As in Fig. 26, the vertical dashed lines in grey indicate the inverse of the eigenvalues of $H^\perp$, $1/h^{(i)}$

#### Central value and covariance of the trained fields

The analytical solution in Eq. (60) is inherently stochastic, since the frozen NTK at $t_\textrm{ref}$ is actually obtained from an ensemble of networks. As a consequence, the operators *U*(*t*) and *V*(*t*) are both random variables. We can then characterize the distribution of the analytical solution using the mean and variance across the ensemble, as shown in Eq. (3). The central value of the analytical solution is thus defined as

61 $$\begin{aligned} \bar{f}_{t,\alpha } = \mathbb {E}\left[ f_{t,\alpha }\right] = \mathbb {E}\left[ U(t)_{\alpha \alpha '} f_{0,\alpha '}\right] + \mathbb {E}\left[ V(t)_{\alpha I} Y_I\right] \, . \end{aligned}$$

More interestingly, we can also compute their covariance matrix at any time *t*,

62 $$\begin{aligned} \textrm{Cov}[f_t,f_t^T]&= \mathbb {E}\left[ U(t) f_0 f_0^T U(t)^T\right] - \mathbb {E}\left[ U(t) f_0\right] \mathbb {E}\left[ f_0^T U(t)^T\right] \nonumber \\&\quad + \mathbb {E}\left[ U(t) f_0 Y^T V(t)^T\right] - \mathbb {E}\left[ U(t) f_0\right] \mathbb {E}\left[ Y^T V(t)^T\right] \nonumber \\&\quad + \mathbb {E}\left[ V(t) Y f_0^T U(t)^T\right] - \mathbb {E}\left[ V(t) Y\right] \mathbb {E}\left[ f_0^T U(t)^T\right] \nonumber \\&\quad + \mathbb {E}\left[ V(t) Y Y^T V(t)^T\right] - \mathbb {E}\left[ V(t) Y\right] \mathbb {E}\left[ Y^T V(t)^T\right] \, . \end{aligned}$$

Note that the first and the fourth lines above yield symmetric matrices, while the third line is just the transpose of the second, thereby ensuring that the whole covariance matrix is the sum of three symmetric matrices and therefore is symmetric,

63 $$\begin{aligned} \textrm{Cov}[f_t,f_t^T] = C_t^{(00)} + C_t^{(0Y)} + C_t^{(YY)}\, , \end{aligned}$$

where

64 $$\begin{aligned} C_t^{(00)}&= \mathbb {E}\left[ U(t) f_0 f_0^T U(t)^T\right] - \mathbb {E}\left[ U(t) f_0\right] \mathbb {E}\left[ f_0^T U(t)^T\right] \, , \end{aligned}$$

65 $$\begin{aligned} C_t^{(0Y)}&= \mathbb {E}\left[ U(t) f_0 Y^T V(t)^T\right] - \mathbb {E}\left[ U(t) f_0\right] \mathbb {E}\left[ Y^T V(t)^T\right] \nonumber \\&\quad + \mathbb {E}\left[ V(t) Y f_0^T U(t)^T\right] - \mathbb {E}\left[ V(t) Y\right] \mathbb {E}\left[ f_0^T U(t)^T\right] \, ,\end{aligned}$$

66 $$\begin{aligned} C_t^{(YY)}&= \mathbb {E}\left[ V(t) Y Y^T V(t)^T\right] - \mathbb {E}\left[ V(t) Y\right] \mathbb {E}\left[ Y^T V(t)^T\right] \, . \end{aligned}$$

Eq. (63) shows explicitly the various contributions to the covariance matrix. Indeed, $C_t^{(00)}$ quantifies the contribution to the covariance matrix that is purely due to the fluctuations of the initial condition, while $C_t^{(YY)}$ quantifies the contribution that is purely due to the statistical fluctuations of the data. The mixed term $C_t^{(0Y)}$ accounts for the correlations between the two sources of uncertainty.

#### Convergence of the analytical solution

We start by comparing the analytical solution (AS), obtained using an ensemble of networks at initialisation as the initial condition, with the trained solution (TS), obtained by training another ensemble of networks drawn from the same prior distribution using GD. This comparison is shown in Fig. 21 for L2 data, where the rows in the grid correspond to different frozen NTKs, while the columns represent numerical and analytical evolution after $T=50, 500$ and 5000 epochs. These results deserve a few comments.

The first observation is that the NTK at early stages of training is not able to drive the prior towards the true function, as shown in the first and, though less dramatically, in the second row of Fig. 21. This is expected, as we extensively discussed in Sect. 3.2.2, since at early stages of training by GD the NTK has not yet aligned its internal representation with the data.

More significantly, we observe a significant discrepancy between the AS and the TS even at $T=5000$. This can be explained as follows. At the beginning of training, there is clearly no difference between AS and TS since both represent the neural network output at initialisation (Sect. 2), with variations due only to different initialisation seeds. During early training stages, AS and TS differ as expected. Indeed, the analytical solution is computed using the frozen NTK at $t_\textrm{ref}$, while the trained solution evolves with an NTK that is still changing as shown in Sect. 3. Crucially, if the NTK at $t_\textrm{ref}$ has already learned from the data and aligned with the solution, the AS converges faster to the target, while the TS requires additional epochs before evolving in the correct direction.

#### Connection with linear methods

We can consider a simplifying limit of Eq. (61), where the initial condition $f_0$ and the data *Y* are statistically independent from the respective evolution operators *U*(*t*) and *V*(*t*). Note that the first term on the right-hand side of Eq. (61) can only be non-zero because of the correlations between *U*(*t*) and $f_0$. In the absence of such correlations, the first term would be given by the product of the expectation values and therefore would vanish if $f_0$ is an ensemble of networks at initialisation. Under these assumptions, we have

67 $$\begin{aligned} \bar{U}(t)&= \mathbb {E}\left[ U(t)\right] \, , \end{aligned}$$

68 $$\begin{aligned} \bar{V}(t)&= \mathbb {E}\left[ V(t)\right] \, , \end{aligned}$$

and

69 $$\begin{aligned} \bar{f}_{t,\alpha } = \bar{U}(t)_{\alpha \alpha '} \bar{f}_{0,\alpha '} + \bar{V}(t)_{\alpha I} Y_I = \bar{V}(t)_{\alpha I} Y_I \,. \end{aligned}$$

The second term in Eq. (61), or equivalently Eq. (69), explicitly shows the contribution of each data point to the central value of the trained fields at each value of $x_{\alpha }$. It is worthwhile remarking that in this limit, the central value from the set of trained networks is a linear combination of the data points, with coefficients given by the evolution operator $V(t)_{\alpha I}$.

In the absence of general theorems, we verify this assumption empirically. From the ensemble of replicas, we generate bootstrap samples and compute the following two estimators,

70 $$\begin{aligned} \Delta [U(t)f_0]&= \mathbb {E}\left[ U(t) f_{0}\right] - \mathbb {E}\left[ U(t) \right] \mathbb {E}\left[ f_{0}\right] \, , \end{aligned}$$

71 $$\begin{aligned} \Delta [V(t)Y]&= \mathbb {E}\left[ V(t) Y\right] - \mathbb {E}\left[ V(t) \right] \mathbb {E}\left[ Y\right] \, . \end{aligned}$$

for different training times, using the same frozen NTK and L2 data. The results are shown in Fig. 22 for the *U* (upper panel) and *V* (lower panel) contributions. The error bands are computed using bootstrap error. By inspecting the figures, we see two distinct patterns emerging. For the operator *U*, $\Delta [U(t) f_0]$ is different from zero for small training times, and thus the correlations between *U*(*t*) and $f_0$ are non-negligible. However, as training proceeds, $\Delta [U(t) f_0]$ becomes compatible with zero within the error bars, suggesting that the correlations are progressively suppressed. The case of the *V* operator is even more striking, as $\Delta [V(t) Y]$ is clearly non-negligible across all training times, although it also shows a decreasing trend as training proceeds. This suggests that the correlations between *V*(*t*) and *Y* cannot be neglected.

Let us conclude this brief discussion by noting that Eq. (69) resembles the structure of a linear method, like Backus–Gilbert or Gaussian Processes. We believe that this observation, as well as the results shown in Fig. 22, deserves further investigations, which we leave for future work. Understanding the relation between different methods is important in order to assess the robustness of the solution.

#### Error decomposition

The analytical expression for the covariance matrix, Eq. (63), allows us to monitor the relative size of the three contributions as training proceeds. For a properly trained ensemble of networks, the covariance of the trained fields should be dominated by the statistical error on the data. We show the diagonal entries of the two contributions $C_t^{(00)}$ (blue band) and $C_t^{(YY)}$ (orange band) to the error budget in Fig. 23, for different frozen NTKs (rows) and different training epochs (columns), using L2 data as before. We do not show the mixed term $C_t^{(0Y)}$ since it is negligible with respect to the other two contributions – the two sources of uncertainty are largely uncorrelated. In general, we observe that towards the end of the training process the contribution from the data $C_t^{(YY)}$ becomes dominant with respect to the contribution coming from the initial condition $C_t^{(00)}$. We also see that the suppression of the initial condition is more severe and happens earlier when the frozen NTK is taken at later stages of training.

In order to study the dependence of the error decomposition on the initial condition, we repeat the same analysis for the case of scaled input $f(x, \log x)$, as described in Sect. 2. Hence, the frozen NTK is taken from an ensemble of trained networks with input layer as in Eq. (8). The initial condition $f_0$ of the analytical solution is drawn from the same prior distribution as the trained solution, and corresponds to the orange curve in Fig. 5. The resulting error decomposition is shown in Fig. 24 for L2 data, where panels are ordered as in Fig. 23. Inspecting the figures, we observe that now the contribution of the initial condition becomes dominant in the small-*x* region, even for large training times and irrespective of the epoch at which the NTK is frozen. This result reflects the behaviour of the prior distribution at small-*x*, where indeed error bands are significantly enlarged with respect to the case of linear input. Interestingly, even the contribution from the data, $C^{(YY)}_t$, increases at small-*x* towards larger training times. This can be explained by observing that, despite not being explicitly dependent on the initial condition $f_0$, the evolution operator *V*(*t*) is constructed from a frozen NTK that has encoded the dependence on the architecture through the training process (see Eq. (102)). That the difference between Figs. 23 and 24 is primarily localised at small-*x* showcases that, for the region left uncovered by the data, the methodology is not able to suppress the dependence on the initial condition. In fact, where the information from the data is available (corresponding roughly to $x \gtrsim 0.01$), the dependence on the initial condition lessens as the analytical solution evolves (left to right). This reduction occurs more rapidly for frozen NTKs taken at later stages of training (top to bottom), showing that there is a non-trivial interplay between the information provided by the data and that acquired by the NTK.

These studies reveal the intricate connection between the prior distribution and the uncertainties of PDFs. In the region constrained by data, the error is dominated by the statistical error on the data, rather than by the fluctuations of the initial fields. This is an important step in our study of the error estimates. It guarantees that the error bars computed from the ensemble of trained PDFs are not biased by the choice of prior, which depends on the selected architecture, activation function, and probability distributions for the biases and weights at initialisation.

### Bias-variance decomposition

Finally, we can use the analytical solution to compute the bias and variance of the trained ensemble of networks. Following the definition of Ref. [4], we define the bias as

72 $$\begin{aligned} \mathcal {B}_t&= \frac{1}{N_\textrm{dat}} \left( \bar{T}[f_t] - Y_\textrm{L0} \right) ^T C_Y^{-1} \left( \bar{T}[f_t] - Y_\textrm{L0} \right) \end{aligned}$$

73 $$\begin{aligned}&= \frac{1}{N_\textrm{dat}} \left( \bar{f_t} - f^\textrm{in}\right) ^T M \left( \bar{f_t} - f^\textrm{in}\right) \end{aligned}$$

where $\bar{T}[f_t] = (\textrm{FK})\bar{f}_t$ is the theoretical prediction computed using the central value of the trained fields at time *t*, Eq. (61), and $Y_\textrm{L0}$ are the data points generated using the input function $f^\textrm{in}$, $Y_\textrm{L0}= (\textrm{FK})f^\textrm{in}$ (see Appendix A). In going from the first to the second line, we use the definition of the matrix *M* given in Eq. (35), which includes the physical information encoded in the FK tables. The bias thus assumes a clear interpretation – it measures the ability of the training process in inverting the forward map used to generate the data. In fact, we recall that the inversion is not exact even with level-0 data; the minimum of the loss is achieved for

74 $$\begin{aligned} f^\textrm{in}_{M^{\perp }} = M^{+} (FK)^T C_Y^{-1} Y_{L0} = M^{+} M f^\textrm{in}\ne f^\textrm{in}, \end{aligned}$$

where $M^{+}$ is the pseudo-inverse of *M*. As in the case of the NTK, there is a residual component of the input vector in the null eigenspace of *M*, $f^\textrm{in}_{M^{\parallel }}$, that is not constrained by the forward map, similarly to the residual noise introduced by the NTK when training the network, Sect. 4.1. Given that this component is not constrained by the data, the parametrization, together with the fitting methodology, has complete freedom to fix it. In our analytical solution, Eq. (60), this freedom is brought in by the linear transformation of the fields $f_0$. On the other hand, $f^\textrm{in}_{M^{\perp }}$ receives its contribution from the data and from the theoretical predictions, resembling the linear transformation of the input data in Eq. (60). This is even more evident if one compares Eq. (74) with Eq. (102)

75 $$\begin{aligned} \mathbb {E} \left[ V(t) Y \right] - f^\textrm{in}_{M^{\perp }}&= \mathbb {E} \left[ \mathcal {M}(t) (\textrm{FK})^TC_Y^{-1} Y \right] \nonumber \\&\quad - M^{+} (\textrm{FK})^TC_Y^{-1} Y_{L0} , \end{aligned}$$

where we recall that *Y* is different from $Y_{L0}$, in general. From this expression, it follows that $\mathcal {M}(t)$ can be seen as a regularised variant of $M^{+}$, with the training time *t* being the regularisation parameter.

These arguments motivate us to reshape Eq. (73) as follows

76 $$\begin{aligned} \mathcal {B}_t = \mathcal {B}_{t,U} + \mathcal {B}_{t,V} + \mathcal {B}_{t,UV} , \end{aligned}$$

where

77 $$\begin{aligned} \mathcal {B}_{t,U}&= \frac{1}{N_\textrm{dat}} \bigl ( \mathbb {E}\left[ U(t)f_0\right] - f^\textrm{in}_{M^{\parallel }} \bigr )^T M \bigl ( \mathbb {E}\left[ U(t)f_0\right] - f^\textrm{in}_{M^{\parallel }} \bigr ) \,, \end{aligned}$$

78 $$\begin{aligned} \mathcal {B}_{t,V}&= \frac{1}{N_\textrm{dat}} \bigl ( \mathbb {E}\left[ V(t)Y\right] - f^\textrm{in}_{M^{\perp }} \bigr )^T M \bigl ( \mathbb {E}\left[ V(t)Y\right] - f^\textrm{in}_{M^{\perp }} \bigr ) \,, \end{aligned}$$

79 $$\begin{aligned} \mathcal {B}_{t,UV}&= \frac{2}{N_\textrm{dat}} \bigl ( \mathbb {E}\left[ U(t)f_0\right] - f^\textrm{in}_{M^{\parallel }} \bigr )^T M \bigl ( \mathbb {E}\left[ V(t)Y\right] - f^\textrm{in}_{M^{\perp }} \bigr ) \,. \end{aligned}$$

Continuing in this vein and following Ref. [4], we define the variance as

80 $$\begin{aligned} \mathcal {V}_t&= \frac{1}{N_\textrm{dat}} \mathbb {E} \biggl [\bigl ( \bar{T}[f_t] - T[f_t] \bigr )^T C_Y^{-1}\bigl ( \bar{T}[f_t] - T[f_t] \bigr )\biggr ] \end{aligned}$$

81 $$\begin{aligned}&= \frac{1}{N_\textrm{dat}} \mathbb {E} \biggl [\bigl ( \bar{f_t} - f_t \bigr )^T M \bigl ( \bar{f_t} - f_t \bigr )\biggr ] \,, \end{aligned}$$

where in the second line we use the same reasoning as in Eq. (73). This expression therefore serves as a measure of the spread of the trained fields in the space of data. Similarly to the bias, we can decompose the variance into three components

82 $$\begin{aligned} \mathcal {V}_t = \mathcal {V}_{t,U} + \mathcal {V}_{t,V} + \mathcal {V}_{t,UV} , \end{aligned}$$

where now the contributions are

83 $$\begin{aligned} \mathcal {V}_{t,U}&= \frac{1}{N_{\textrm{dat}}} \mathbb {E} \biggl [ \bigl ( \mathbb {E} [U(t)f_0] - U(t)f_0 \bigr )^T M \bigl ( \mathbb {E} [U(t)f_0] - U(t)f_0 \bigr ) \biggr ]\,, \end{aligned}$$

84 $$\begin{aligned} \mathcal {V}_{t,V}&= \frac{1}{N_{\textrm{dat}}} \mathbb {E} \biggl [ \bigl ( \mathbb {E} [V(t)Y] - V(t)Y \bigr )^T M \bigl ( \mathbb {E} [V(t)Y] - V(t)Y \bigr ) \biggr ]\,, \end{aligned}$$

85 $$\begin{aligned} \mathcal {V}_{t,UV}&= \frac{2}{N_{\textrm{dat}}} \mathbb {E} \biggl [ \bigl ( \mathbb {E} [U(t)f_0] - U(t)f_0 \bigr )^T M \bigl ( \mathbb {E} [V(t)Y] - V(t)Y \bigr ) \biggr ]\,. \end{aligned}$$

Bias and variance, together with their decompositions introduced above, are shown in Figs. 25 and 26, respectively. We show these curves as functions of the training time for different frozen NTKs and for L0, L1 and L2 data. We now discuss these figures in turn.

Starting from the bias, we see that $\mathcal {B}_{t,V}$ contributes the most to the total budget of the bias, in accordance with the observations made in Fig. 20. Furthermore, the bias decreases more rapidly when the NTK is frozen at later stages of training, as the NTK has had more time to align with the data and therefore is more effective in reducing the bias. We also observe that the behaviour of the bias at larger epochs is different depending on the closure level. For L0 data, the bias decreases monotonically with training time, approaching zero asymptotically. This is expected, since L0 data do not contain any noise and the bias can be reduced indefinitely by training longer. On the other hand, the bias for L1 and L2 data presents a minimum at large epochs, after which the bias increases again (see the inset in each figure). Here the dependence on the frozen NTK determines the location of the minimum – the later the NTK is frozen, the earlier the bias reaches its minimum. That both L1 and L2 have a similar behaviour tells us that the central value of the analytical solution is mostly insensitive to the experimental uncertainty propagated through the Monte Carlo fluctuations of the data.

Turning now to the variance, we see again that the dominant contribution comes from $\mathcal {V}_{t,V}$, unless the NTK is frozen at very early stages of training. This agrees with Fig. 23, where we observed that the *U* contribution is significant only at early epochs. In general, the variance exhibits a peak at intermediate epochs, after which it decreases. This stands out particularly for $T_\textrm{ref} = 3000$ (second column in Fig. 26), where the height of the peak has its maximum for the case of L2 data. When the NTK is frozen at later stages of training, the peak is less pronounced and located at earlier epochs. Note that, contrary to the bias, the behaviour at large epochs of the variance differs between L1 and L2 data. In fact, while the variance for L0 and L1 data approaches zero asymptotically, for L2 data the variance converges to a residual value shifted from zero. Finally, a closer inspection of the figures reveals a strong correlation between the eigenvalues of $H^\perp$, introduced in Sect. 4.1, and the transitions in the slope of the variance. Indeed, the components of the analytical solution contribute with different timescales, characterised by the inverse of the eigenvalues of $H^\perp$. This is particularly evident for the case at $T_\textrm{ref}=5000$, where in correspondence of the timescale $1/h^{(i)}$, shown as vertical dashed lines in grey, the variance shows a change in slope. This remains true across all closure-test data, although slightly mitigated for L2 data.

The interplay between bias and variance, as well as the presence of extrema in both curves, has practical implications in the choice of the optimal stopping time. In Fig. 27 we compare the total contributions of bias and variance as functions of the training time, for different frozen NTKs, and for L0, L1 and L2 data. One major observation that emerges from these figures is that the intersection point between bias and variance – which defines a bias-variance ratio equal to one – is not sufficient to determine whether the fit has converged optimally. For instance, for L0 data with the NTK frozen at $T_\textrm{ref}=3000$ (first row, third column), the two curves intersect at around $T \sim 1000$ and $T> 10000$. Clearly, only the latter represents a good fit. This becomes even more evident for L2 data (third row), in particular when the NTK is frozen at $T_\textrm{ref}=10000$ (last column). Interestingly, there are no intersection points between bias and variance for L0 data and when the NTKs are frozen at $T_\textrm{ref} > 3000$. This is a direct consequence of the observations made above, namely that the bias is insensitive to the noise in the data, while the variance is. Indeed, as is clear by comparing L1 and L2 data for $T_\textrm{ref} = 10000$, adding the Monte Carlo fluctuations in the data leaves the bias almost unchanged but increases the variance, resulting in the intersection of the two curves.

We conclude by remarking that bias and variance are computed using the analytical solution in Eq. (60), hence from an ensemble of frozen NTKs. The results discussed above provide evidence that the NTK is capable of encoding not only the physical features of the data, but also the statistical features of the methodology. Such a finding deserves further attention in future assessments of PDF determinations.

## Conclusions

Our present age is marked by unprecedented advancements in machine learning techniques, whose applications span many scientific domains – PDF determination being one of them. It is of paramount importance to understand how these new techniques behave when applied to complex problems such as PDF determination.

In this work, we have taken a step forward in this direction. We have investigated a novel treatment of the learning process in the context of PDF fitting by exploring the training dynamics in the functional space of the neural network. In fact, the NTK can be used to unravel complex dynamics obfuscated by the training algorithms commonly employed in PDF fits. We have shown that the properties of the NTK are highly entangled with the fitting results, and that a proper understanding of its structure and time dependence can provide precious insights on the learning process. The identification of separate rich and lazy training phases is potentially a general feature of MLP training, which is worthy of highlighting beyond the specific application to PDF fits. In the context of PDF fits, we have developed, under certain assumptions, an analytical description of how the neural network evolves during training, enabling us to better understand the NNPDF methodology and its dependence on the underlying model architecture. To the best of our knowledge, such description has not yet been attempted in the context of ill-defined inverse problems.

Yet, this work is far from being conclusive. As a pioneering study, we believe that the most important contribution is the identification and initial discussion of the role of the NTK in PDF fits, leading to a set of relevant diagnostic metrics to be explored in future works. Indeed, many aspects merit further investigations. First of all, we need to study in detail the outcome of a similar analysis when applied to a more complex and realistic framework, including multiple fitted flavors and real data. For data that depend linearly on the PDFs, the extension of the formalism is straightforward. However, the agreement with the analytical predictions need to be tested by performing the numerical simulations. Further insights could be gained by exploring different parametrizations beyond neural networks. These systematic studies will be carried out in forthcoming works using the recent flexible and extensible open-source framework Colibri [34].

In spite of the simplified framework adopted in the present study, our findings highlight the complexity and richness of the learning process; a quantitative description of the learning process is needed in order to pin down the origin of PDF uncertainties. This poses a significant challenge in light of the improved precision of forthcoming measurements. We believe that the tools presented here can help address this gap.

## Figures and Tables

**Fig. 28:**
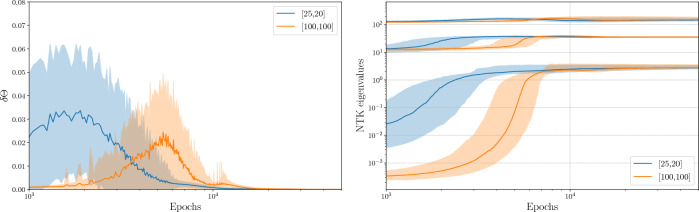
Comparison of the variation of the NTK during training (left) and the first three eigenvalues (right) for two different architectures with sizes [28, 20] and [100, 100] respectively. In both cases, L0 data is used. In the left plot, error bands represent the standard deviation over the ensemble of replicas. In the right plot, solid lines represent the median over the ensemble of networks, while solid bands correspond to 68% confidence level

**Fig. 29:**
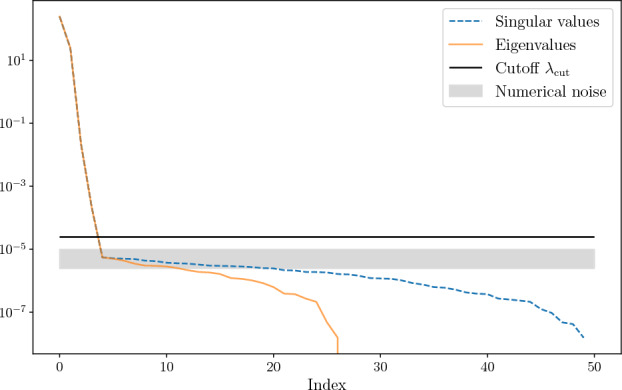
Visualisation of the cut-off tolerance for the NTK spectrum used in Sect. 4. Eigenvalues (orange solid line) and singular values (blue dashed line) are shown, together with the cut-off tolerance (black) chosen as described in the text. We also show the corresponding numerical noise (grey shaded area) which occurs at small eigenvalues (or singular values)

**Fig. 30:**
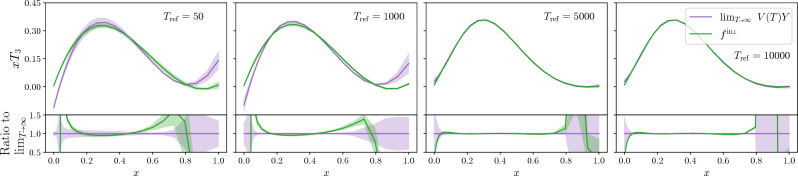
Test of the $t\rightarrow \infty$ limit of the L0 training for different frozen NTKs. The green curve represents the projection of the input function $f^\textrm{in}$ onto the subspace orthogonal to the kernel of the NTK at $t_\textrm{ref}$, i.e., $f^\mathrm{in \perp }$. The purple curve represents the contribution of the operator *V*, computed with the NTK at $T_\textrm{ref}$, in the limit of infinite training time

**Fig. 31:**
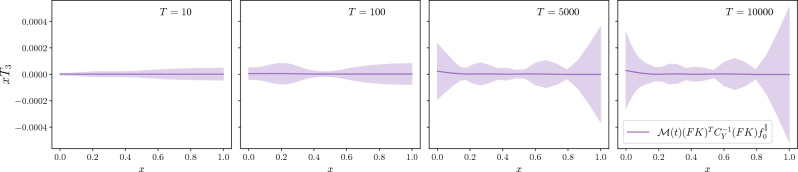
Test of the average of the parallel contribution for different epochs. The reference epoch at which the frozen NTK is chosen is $T_\textrm{ref} = 10000$. L2 data is used in the plot. Note that the scale on the vertical axis is three orders of magnitude smaller than in Fig. 30

## Data Availability

This manuscript has associated data in a data repository. [Authors’ comment: The data generated and analysed in the present work, are available in the GitHub repository https://github.com/achiefa/DeepLearningTheory.]

## The BCDMS dataset for $T_3$

The analysis presented in this work employs a set of synthetic data points generated using a known underlying law $f^\textrm{in}$ that we seek to reconstruct. Analogous to Ref. [2], the pseudo-data are constructed by convolving $f^\textrm{in}$ with FK tables whose kinematic dependence is specified by measurements of the structure function $F_2$ on proton and deuterium targets from the BCDMS collaboration [35]. By combining these measurements, one can construct the observable $F_2^p - F_2^d$ that, at next-to-next-to-leading order (NNLO) in QCD and under the assumption of isoscalarity for the deuterium nucleus, provides a clean probe of the non-singlet triplet PDF combination $T_3 = u^+ - d^+$, where $u^+ = u + \bar{u}$ and $d^+ = d + \bar{d}$. The factorisation formula in Eq. (1) then simplifies to

86 $$\begin{aligned} F_2^p - F_2^d = C_{T_3} \otimes T_3, \end{aligned}$$

where $C_{T_3}$ is the corresponding Wilson coefficient computed in perturbative QCD, and $\otimes$ is a short-hand notation that denotes the convolution as in Eq. (1). The construction of the FK tables needed to compute the predictions, as well as the construction of the covariance matrix, are identical to Ref. [2], to which the reader is referred for further details. This yields a total of 333 data points, which then reduce to 248 points after applying the kinematic cuts.

Following the closure test framework developed by the NNPDF collaboration [1, 4], we generate data with three different levels of noise, labelled as Level 0 (L0), Level 1 (L1) and Level 2 (L2) data. Furthermore, we use the non-singlet triplet $xT_3$ from the NNPDF4.0 parton set [4] as the input law $f^\textrm{in}$. In the following, we summarise the definition of the different levels of pseudo-data.

**Level 0** The pseudo-data are generated without any experimental noise, i.e., by using the input function and the FK tables as follows

87 $$\begin{aligned} Y_{L0} = (\textrm{FK})f^\textrm{in}. \end{aligned}$$

In this ideal scenario, the analysis should reproduce the input $f^\textrm{in}$, though some residual reconstruction error may remain in the kinematic region not covered by the FK tables. Level 0 assesses the intrinsic bias of the methodology, as any neural network replica will be trained on the same data points $Y_{L0}$.

**Level 1** In this case, the experimental noise is added on top of the L0 data, by sampling from the multivariate normal distribution with the full experimental covariance matrix $C_Y$ provided by the BCDMS collaboration

88 $$\begin{aligned} Y_{L1} = Y_{L0} + \eta , \quad \text {where} \quad \eta \sim \mathcal {N}(0, C_Y). \end{aligned}$$

This case is closer to actual experimental data, where the “true” value is blurred by the presence of noise. Note however that we are not yet propagating the experimental uncertainties into the uncertainties of the fitted PDF, as the added noise is fixed over all replicas.

**Level 2** Finally, we generate L2 pseudo-data by adding a different noise realisation to each replica, sampled from the same multivariate normal distribution

89 $$\begin{aligned} Y_{L2}^{(k)} = Y_{L1} + \xi ^{(k)}, \quad \text {where} \quad \xi ^{(k)} \sim \mathcal {N}(0, C_Y). \end{aligned}$$

This represents the most realistic scenario where both model and data uncertainties are present. In this case, each neural network replica will be trained on a different set of data points $Y_{L2}^{(k)}$.

## Dependence on the architecture of the NTK

It is interesting to consider what happens to the picture sketched so far as the architecture of the neural networks is varied. In Fig. 28 we compare the time dependence of the Frobenius norm of the NTK and the variation of the first three eigenvalues for two different architectures. The smaller network is the [28, 20] used in the standard NNPDF analyses and in this work, while the large one is a [100, 100] network, which is closer to the infinite-width limit. For illustration purposes we focus in this plot on L1 data and only three eigenvalues rather than the five we examined above. The quantitative features are exactly the same for L0 and L2 data, and adding the fourth and fifth eigenvalues does not add unexpected behaviours compared to what we observe in Fig. 8. It is interesting to remark that the onset of lazy training is slower for the larger network. This is to be expected if we interpret the early stages of training as a phase where the network identifies the learnable features in the space of functions that it can parametrize. For a larger network, the space of parametrized functions is larger and the identification of the physical features takes a larger number of epochs.

## Cut-off tolerance of the NTK spectrum

We determine the effective rank of the NTK by identifying eigenvalues that are numerically significant. We classify eigenvalues $\lambda _i$ as numerically zero if

90 $$\begin{aligned} \lambda _i < \epsilon _\textrm{tol} \cdot \lambda _\textrm{max} , \end{aligned}$$

where $\epsilon _\textrm{tol}$ is the relative tolerance and $\lambda _\textrm{max}$ is the largest eigenvalue of the NTK. Throughout this work, we use $\epsilon _\textrm{tol} = 10^{-7}$, which is close to machine precision for single-precision floating point numbers. This choice is illustrated in Fig. 29, where we show the NTK spectrum at initialization for the NNPDF-like architecture discussed in the main text (orange solid line). Together with the eigenvalues, we also show the singular values of the NTK (dashed blue line) to illustrate that the two spectra are identical until numerical noise sets in. The cut-off tolerance, indicated by the black horizontal line, is chosen to be slightly above the numerical noise level (grey shaded area) to ensure that only numerically significant eigenvalues are retained.

## Detailed derivation of the analytical solution

The solution to Eq. (56) can be written as the sum of the solution of the homogeneous equation, $\hat{f}^{\perp }_{t,k}$, and a particular solution of the full equation. The solution of the homogeneous equation is

91 $$\begin{aligned} \hat{f}^{\perp }_{t,k} = \sum _{i=1}^{d^\perp } f^{\perp (i)}_{0} e^{-h^{(i)}t} w^{(i)}_k\, , \end{aligned}$$

where

92 $$\begin{aligned} f^{\perp (i)}_{0} = \sum _{k=1}^{d_\perp } w^{(i)}_k f^\perp _{0,k}\, , \end{aligned}$$

guarantees that the initial condition $\hat{f}^\perp _{t,k}=f^\perp _{0,k}$ is satisfied. Similarly, if we define

93 $$\begin{aligned} \Upsilon ^{(i)} = \sum _{k=1}^{d_\perp } w^{(i)}_k B^\perp _{k}\, , \end{aligned}$$

then

94 $$\begin{aligned} \check{f}^\perp _{t,k} = \mathop {{\mathop {\sum }\nolimits '}}\limits _{i} \frac{1}{h^{(i)}} \Upsilon ^{(i)} \left( 1 - e^{-h^{(i)}t}\right) w^{(i)}_k\, , \end{aligned}$$

where the sum only involves the non-zero modes of $H^\perp$, is a particular solution of the inhomogeneous equation, which satisfies the boundary condition $\check{f}^{\perp }_{0,k}=0$. Hence, the solution of the flow equation in the subspace orthogonal to $\text {ker}\ \Theta$ is

95 $$\begin{aligned} f^\perp _{t,k}&= \hat{f}^\perp _{t,k} + \check{f}^\perp _{t,k}\, . \end{aligned}$$

Finally, collecting the parallel contribution, Eq. (55), and the solution of the orthogonal component, Eq. (95), yields a simple expression,

96 $$\begin{aligned} f_{t,\alpha } = U(t)_{\alpha \alpha '} f_{0,\alpha '} + V(t)_{\alpha I} Y_{I}\, . \end{aligned}$$

The two evolution operators *U*(*t*) and *V*(*t*) are here summarised:

97 $$\begin{aligned} U(t)_{\alpha \alpha '} = \hat{U}^\perp (t)_{\alpha \alpha '} + \check{U}^\perp (t)_{\alpha \alpha '} + U^\parallel _{\alpha \alpha '}\, , \end{aligned}$$

where

98 $$\begin{aligned} \hat{U}^\perp (t)_{\alpha \alpha '} = \sum _{k,k'\in \perp } \sqrt{\lambda ^{(k)}} z^{(k)}_\alpha \left[ \sum _i w^{(i)}_{k} e^{-h^{(i)}t} w^{(i)}_{k'}\right] z^{(k')}_{\alpha '} \frac{1}{\sqrt{\lambda ^{(k')}}}\, , \end{aligned}$$

and

99 $$\begin{aligned} U^\parallel _{\alpha \alpha '} = \sum _{k''\in \parallel } z^{(k)}_\alpha z^{(k)}_{\alpha '} \, . \end{aligned}$$

The contributions from $\check{U}^\perp (t)$ and *V*(*t*) are more easily expressed by introducing the operator

100 $$\begin{aligned} \mathcal {M}(t)_{\alpha \alpha '}&= \sum _{k,k'\in \perp } \sqrt{\lambda ^{(k)}} z^{(k)}_\alpha \left[ \mathop {{\mathop {\sum }\nolimits '}}\limits _{i} w^{(i)}_{k} \frac{1}{h^{(i)}}\, \left( 1- e^{-h^{(i)}t}\right) w^{(i)}_{k'}\right] \nonumber \\&\quad z^{(k')}_{\alpha '} \sqrt{\lambda ^{(k')}}\,. \end{aligned}$$

Then, we can write

101 $$\begin{aligned} \check{U}^\perp (t) = - \mathcal {M}(t)\; (\textrm{FK})^TC_Y^{-1} (\textrm{FK})\left[ \sum _{k''\in \parallel } z^{(k'')} z^{(k'') T}\right] \, , \end{aligned}$$

and

102 $$\begin{aligned} V(t) = \mathcal {M}(t)\; (\textrm{FK})^TC_Y^{-1}\, , \end{aligned}$$

where we note that the term in the bracket in Eq. (101) is simply the projector on the kernel of the NTK. The four terms that appear in the analytical solution have a clear physical interpretation:

- The first term $\hat{U}^\perp (t)$ suppresses the components of the initial condition that lie in the subspace orthogonal to the kernel of the NTK. These are the components that are learned by the network during training. While the trained solution still depends on its value at initialisation, that dependence is suppressed during training. This suppression is exponential in the training time, and the rates are given by the eigenvalues of $H^{\perp }$.
- The contribution from $U^\parallel$ yields the component of the initial condition that lies in the kernel of the NTK. As such, those components remain unchanged during training and are part of the trained field at all times *t*.
- The two remaining contributions are best understood by combining them together,
  103 $$\begin{aligned} \check{U}^{\perp }(t) f_{0} + V(t) Y = \mathcal {M}(t)\; (\textrm{FK})^TC_Y^{-1} \left[ Y - (\textrm{FK})f_{0}^{\parallel }\right] \, . \end{aligned}$$
  The parallel component of the initial condition $f_{0}^{\parallel }$ does not evolve during training, and therefore it yields a contribution $(\textrm{FK})f_{0}^{\parallel }$ to the theoretical prediction of the data points at all times *t*. This is taken into account by subtracting this contribution from the data, before the inference is performed.

### Crosschecks using L0 data

The analytical solution enables rigorous validation of our implementation through crosschecks with L0 data, where we have complete control over the data generation process. In this case, the realization of the dataset is completely determined by the input PDFs

104 $$\begin{aligned} Y = (\textrm{FK})f^\textrm{in}\,. \end{aligned}$$

Note that using L0 data only affects the second term in Eq. (60).^13^ We can then rewrite the combined term in Eq. (103) as follows

105 $$\begin{aligned} \check{U}^\perp (t) f_0 + V(t) Y&= \mathcal {M}(t)\, (\textrm{FK})^TC_{Y}^{-1} (\textrm{FK})\, \left[ f^\textrm{in}- f_{0}^\parallel \right] \, . \end{aligned}$$

The subtraction taking place in the square brackets of Eq. (105) suggests that the effective function that the neural network actually sees is not the input function $f^\textrm{in}$ used to generate the data, but rather the difference between $f^\textrm{in}$ and the component of the initial function $f_0$ that lies in the subspace spanned by the kernel of the NTK, i.e., $f_0^\parallel$. In other words, the parallel component $f_0^\parallel$, which, we remind the reader, does not evolve during the analytic training, acts as a constant “bias” in the training process, effectively shifting the input function seen by the neural network. Of course the actual magnitude of this irreducible noise depends both on how $f_0$ and the kernel of the NTK are distributed over the ensemble.

Note that the observation above remains true even in the limit of infinite training,

106 $$\begin{aligned} \lim _{t\rightarrow \infty } V(t) Y = f^\mathrm{in \perp }+ \mathcal {M}_{\infty } M f^\mathrm{in \parallel }\, , \end{aligned}$$

which shows that the *V* component of the trained solution reproduces exactly the component of the PDF that lies in the subspace orthogonal to the kernel of $\Theta$. We compare the asymptotic behaviour of *V*(*t*)*Y* and $f^\mathrm{in \perp }$ in Fig. 30, where we see that the analytical solution at infinite training time reproduces the expected result, i.e., it coincides with $f^\mathrm{in \perp }$, as long as $T_{\textrm{ref}}> 1000$. The second term in the square bracket on the right-hand side of Eq. (105) is the contribution from the parallel component at initialisation that does not evolve in the training process. Given that $f_0$ is almost normally distributed around zero, that term does not contribute to the central value of the fitted PDF, i.e., to the average of the trained solution over replicas.

The time evolution of

107 $$\begin{aligned} \mathbb {E}\left[ \mathcal {M}(t)\, (\textrm{FK})^TC_{Y}^{-1} (\textrm{FK})\, f_{0}^\parallel \right] \, , \end{aligned}$$

is shown in Fig. 31.

### Infinite training time

In the limit of infinite training time, the evolution operators *U*(*t*) and *V*(*t*) simplify and yield an elegant interpretation of the minimum of the cost function. For large training times, we have

108 $$\begin{aligned}&\hat{U}^\perp _{\infty , \alpha \alpha '} = \lim _{t\rightarrow \infty }\hat{U}^\perp (t)_{\alpha \alpha '} = 0\, \end{aligned}$$

109 $$\begin{aligned}&\mathcal {M}_{\infty , \alpha \alpha '} \quad = \lim _{t\rightarrow \infty }\mathcal {M}(t)_{\alpha \alpha '} = \sum _{k,k'\in \perp } \sqrt{\lambda ^{(k)}} z^{(k)}_\alpha \nonumber \\&\qquad \left[ \mathop {{\mathop {\sum }\nolimits '}}\limits _{i} w^{(i)}_{k} \frac{1}{h^{(i)}}\, w^{(i)}_{k'}\right] z^{(k')}_{\alpha '} \sqrt{\lambda ^{(k')}}\, , \end{aligned}$$

and explicit expressions for $\check{U}^\perp _{\infty }$ and $V_{\infty }$ are obtained from $\mathcal {M}_{\infty }$. The term in the square bracket in Eq. (109) is the spectral decomposition of the pseudo-inverse of $H^\perp$ in $d_\perp$-dimensional orthogonal subspace. So, the operator $\mathcal {M}_{\infty }$ acts as follows on a field $f_{\alpha }$:

1. The term on the right of the square bracket computes the coordinate $f_{k'}$ introduced in Eq. (48). The $f_k$ are the coordinates of the component $f^\perp$ that evolves during training,
  110 $$\begin{aligned} f^\perp = \sum _{k\in \perp } \sqrt{\lambda ^{(k)}} f_k\, z^{(k)}\, . \end{aligned}$$
2. The term in the square bracket applies the pseudo-inverse to the coordinates $f_k$,
  111 $$\begin{aligned} f'_k = \left( H^\perp \right) ^+_{kk'} f_{k'}\, . \end{aligned}$$
3. The final term on the left of the square bracket reconstructs the full field corresponding to the coordinates $f'_{k}$,
  112 $$\begin{aligned} f^{'\perp } = \sum _{k\in \perp } \sqrt{\lambda ^{(k)}} f'_{k}\, z^{(k)}\, . \end{aligned}$$

As discussed at the end of Sect. 4.1 it is convenient to combine the contributions of $\check{U}^\perp _{\infty }$ and $V_{\infty }$,

113 $$\begin{aligned} \check{U}^{\perp }_{\infty } f_{0} + V_{\infty } Y = \mathcal {M}_{\infty }\; (\textrm{FK})^TC_Y^{-1} \left[ Y - (\textrm{FK})f_{0}^{\parallel }\right] \, . \end{aligned}$$

The contribution to the observables from the parallel components of *f* does not change during training, therefore that contribution is subtracted from the data and the orthogonal components of *f* are adjusted to minimize the $\chi ^2$ of the corrected data. The minimum of the $\chi ^2$ in the orthogonal subspace is found applying $\mathcal {M}_{\infty }$, i.e., by projecting in the orthogonal subspace, applying the pseudo-inverse and finally recomputing the full field as detailed above.

## Footnotes

1 When omitting the data index *I*, we will always assume $Y \in \mathbb {R}^{N_{\textrm{dat}}}$.

2 Following the same convention used for the data, when omitting the grid index $\alpha$, and/or the flavor index *i*, we will always refer to a vector $f \in \mathbb {R}^{N_{f}\times N_{\textrm{grid}}}$.

3 We refer to *i* as the *neuron* index.

4 For simplicity, we ignore the preprocessing function $x^{1-\delta _i} (1-x)^{\eta _i}$ that is currently used in the NNPDF fits. While the preprocessing may be useful in speeding the training it does not affect the current discussion.

5 We have denoted *all* couplings by $\gamma ^{{(\ell )}}$. Different couplings are identified by the number of indices, so that $\gamma ^{(\ell )}_{\alpha _1\alpha _2}$ is a two-point coupling, $\gamma ^{(\ell )}_{\alpha _1\alpha _2,\alpha _3\alpha _4}$ is a four-point coupling, etc.

6 The notation here refers to the matrix element $(\alpha _1,\alpha _2)$ of the inverse matrix of $\gamma ^{(\ell )}$, and *not* to the inverse of the matrix element $\gamma ^{(\ell )}_{\alpha _1\alpha _2}$.

7 Note that, in addition to the scaling $\mathcal {O}(1/n)$ theoretically predicted for large networks, the uncertainty bands include bootstrap errors due to the finite size of the ensemble. Using an ensemble of 100 replicas, the bootstrap error on the standard deviation is $\sim 10\%$.

8 Note that, due to the large difference in magnitude of the eigenvalues, the finite precision used in our codes introduces noise in the decomposition, so that small eigenvalues should be effectively considered zero. We discuss the cut-off tolerance in Appendix C.

9 Geometrically, *M* is the metric that measures how distinguishable two models are in terms of their predictions. In fact, using L0 data the loss can be written as $\mathcal {L} = \tfrac{1}{2}(f^\textrm{in}- f)^T M (f^\textrm{in}- f)$: two PDFs are *indistinguishable* when they differ by a vector in $\ker M$.

10 In the ML literature, the basis functions $\phi _i(x)$ are often referred to as features.

11 Despite this result having been obtained using the frozen NTK, it is worth mentioning that at any time during training the kernel of the NTK is always defined and in general non-empty. Hence, even in the initial stages of the training process, there is a component that is completely determined by the initial condition, i.e., by the prior distribution in functional space.

12 Unless stated otherwise, in this section central values and uncertainties are always computed as ensemble averages across replicas.

## Code Availability Statement

This manuscript has associated code/software in a data repository. [Authors’ comment: The code used in the analysis, are available in the GitHub repository https://github.com/achiefa/DeepLearningTheory.]

## References

[1] L. Del Debbio, T. Giani, M. Wilson, Bayesian approach to inverse problems: An application to NNPDF closure testing. Eur. Phys. J. C **82**(4), 330 (2022). arXiv:2111.05787

[2] A. Candido, L. Del Debbio, T. Giani, G. Petrillo, Bayesian inference with Gaussian processes for the determination of parton distribution functions. Eur. Phys. J. C **84**(7), 716 (2024). arXiv:2404.07573

[3] Y.C. Medrano, H. Dutrieux, J. Karpie, K. Orginos, S. Zafeiropoulos, Gaussian processes for inferring parton distributions. arXiv:2510.21041

[4] NNPDF Collaboration, R.D. Ball et al., The path to proton structure at 1% accuracy. Eur. Phys. J. C **82**(5), 428 (2022). arXiv:2109.02653

[5] A. Ablat et al., New results in the CTEQ-TEA global analysis of parton distributions in the nucleon. Eur. Phys. J. Plus **139**(11), 1063 (2024). arXiv:2406.10260

[6] S. Bailey, T. Cridge, L.A. Harland-Lang, A.D. Martin, R.S. Thorne, Parton distributions from LHC, HERA, Tevatron and fixed target data: MSHT20 PDFs. Eur. Phys. J. C **81**(4), 341 (2021). arXiv:2012.04684

[7] S. Alekhin, J. Blümlein, S. Moch, R. Placakyte, Parton distribution functions, , and heavy-quark masses for LHC Run II. Phys. Rev. D **96**(1), 014011 (2017). arXiv:1701.05838

[8] A. Chiefa, M.N. Costantini, J. Cruz-Martinez, E.R. Nocera, T.R. Rabemananjara, J. Rojo, T. Sharma, R. Stegeman, M. Ubiali, Parton distributions confront LHC Run II data: a quantitative appraisal. JHEP **07**, 067 (2025). arXiv:2501.10359

[9] L.A. Harland-Lang, T. Cridge, R.S. Thorne, A stress test of global PDF fits: closure testing the MSHT PDFs and a first direct comparison to the neural net approach. Eur. Phys. J. C **85**(3), 316 (2025). arXiv:2407.07944 40115426 10.1140/epjc/s10052-025-13934-3 PMC11920009

[10] PDF4LHC Working Group Collaboration, R.D. Ball et al., The PDF4LHC21 combination of global PDF fits for the LHC Run III. J. Phys. G **49**(8), 080501 (2022). arXiv:2203.05506

[11] ATLAS Collaboration, G. Aad et al., A precise determination of the strong-coupling constant from the recoil of bosons with the ATLAS experiment at TeV. arXiv:2309.12986

[12] CMS Collaboration, A. Hayrapetyan et al., Measurement of the Drell–Yan forward–backward asymmetry and of the effective leptonic weak mixing angle in proton-proton collisions at s = 13 TeV. Phys. Lett. B **866**, 139526 (2025). arXiv:2408.07622

[13] ATLAS Collaboration. Measurement of the W-boson mass and width with the ATLAS detector using proton–proton collisions at TeV. Eur. Phys. J. C **84**(12), 1309 (2024). 10.1140/epjc/s10052-024-13190-x

[14] V. Chekhovsky et al., High-precision measurement of the W boson mass with the CMS experiment. Nature **652**(8109), 321–327 (2026). arXiv:2412.13872 41951965 10.1038/s41586-026-10168-5 PMC13061639

[15] A. Barontini, M.N. Costantini, G. De Crescenzo, S. Forte, M. Ubiali, Evaluating the faithfulness of PDF uncertainties in the presence of inconsistent data. arXiv:2503.17447

[16] J. Cruz-Martinez, S. Forte, E.R. Nocera, Future tests of parton distributions. Acta Phys. Polon. B **52**, 243 (2021). arXiv:2103.08606

[17] A. Jacot, F. Gabriel, C. Hongler, Neural tangent kernel: convergence and generalization in neural networks. Adv. Neural. Inf. Process. Syst. **31**, 1 (2018). arXiv:1806.07572

[18] S. Tovey, S. Krippendorf, M. Spannowsky, K. Nikolaou, C. Holm, Collective variables of neural networks: empirical time evolution and scaling laws. Mach. Learn. Sci. Technol. **6**(3), 035021 (2025). arXiv:2410.07451

[19] J. Lee, L. Xiao, S. Schoenholz, Y. Bahri, R. Novak, J. Sohl-Dickstein, J. Pennington, Wide neural networks of any depth evolve as linear models under gradient descent. J. Stat. Mech: Theory Exp. **2020**(12), 124002 (2020). arXiv:1902.06720

[20] D.A. Roberts, S. Yaida, B. Hanin, *The Principles of Deep Learning Theory* (Cambridge University Press, Cambridge, 2022), p.5

[21] X. Glorot, Y. Bengio, Understanding the difficulty of training deep feedforward neural networks. in Proceedings of the Thirteenth International Conference on Artificial Intelligence and Statistics, pp. 249–256, JMLR Workshop and Conference Proceedings (2010)

[22] M.N. Costantini, M. Madigan, L. Mantani, J.M. Moore, A critical study of the Monte Carlo replica method. JHEP **12**, 064 (2024). arXiv:2404.10056

[23] A. Maiti, K. Stoner, J. Halverson, Symmetry-via-duality: invariant neural network densities from parameter-space correlators. arXiv:2106.00694

[24] A. Chiefa, L.D. Debbio, R. Kenway, In preparation

[25] S. ichi Amari, Backpropagation and stochastic gradient descent method. Neurocomputing **5**(4), 185–196 (1993)

[26] T. Dozat, Incorporating Nesterov momentum into Adam. in Proceedings of the 4th International Conference on Learning Representations, pp. 1–4 (2016)

[27] D.P. Kingma, J. Ba, Adam: a method for stochastic optimization. arXiv:1412.6980

[28] M.N. Costantini, L. Mantani, J.M. Moore, M. Ubiali, A linear PDF model for Bayesian inference. arXiv:2507.16913

[29] D.G.T. Barrett, B. Dherin, Implicit gradient regularization. arXiv:2009.11162

[30] S. Arora, S.S. Du, W. Hu, Z. Li, R.R. Salakhutdinov, R. Wang, On exact computation with an infinitely wide neural net. arXiv:1904.11955

[31] S. Alemohammad, Z. Wang, R. Balestriero, R. Baraniuk, Recurrent neural tangent kernels: adding memory to neural tangent kernels. arXiv:2006.10246

[32] NNPDF Collaboration, R.D. Ball et al., Parton distributions for the LHC Run II. JHEP **04**, 040 (2015). arXiv:1410.8849

[33] S. Fort, G.K. Dziugaite, M. Paul, S. Kharaghani, D.M. Roy, S. Ganguli, Deep learning versus kernel learning: an empirical study of loss landscape geometry and the time evolution of the neural tangent kernel. Adv. Neural. Inf. Process. Syst. **33**, 5850–5861 (2020). arXiv:2010.15110

[34] M.N. Costantini, L. Mantani, J.M. Moore, V.S. Sanchez, M. Ubiali, Colibri: a new tool for fast-flying PDF fits. arXiv:2510.03391

[35] A.C. Benvenuti, A high statistics measurement of the proton structure functions and from deep inelastic muon scattering at high . Phys. Lett. B **223**, 485 (1989)

